# Modifying the Glycocalyx of Melanoma Cells via Metabolic Glycoengineering Using *N*-Acetyl-d-glucosamine Analogues

**DOI:** 10.3390/cells13221831

**Published:** 2024-11-06

**Authors:** David Harris, Marcel Groß, Sebastian Staebler, Regina Ebert, Jürgen Seibel, Anja Katrin Boßerhoff

**Affiliations:** 1 Institute of Biochemistry, Friedrich Alexander University Erlangen-Nürnberg, Fahrstrasse 17, 91054 Erlangen, Germany; david.harris@fau.de (D.H.); sebastian.staebler@fau.de (S.S.); 2 Institute of Organic Chemistry, Julius-Maximilians-University of Würzburg, Am Hubland, 97074 Würzburg, Germany; marcel.gross@uni-wuerzburg.de; 3 Department of Musculoskeletal Tissue Regeneration, Orthopedic Clinic König-Ludwig Haus, Julius-Maximilians-University of Würzburg, Friedrich-Bergius-Ring 15, 97076 Würzburg, Germany; regina.ebert@uni-wuerzburg.de

**Keywords:** click chemistry, glycoproteins, melanoma cells, metabolic glycoengineering

## Abstract

Tumor cells are decorated with aberrant glycan structures on cell surfaces. It is well known that the glycocalyx serves as a main cellular regulator, although its role in cancer is still not completely understood. Over recent decades, several non-natural monosaccharides carrying clickable groups have been introduced in melanoma cells. This technique, called Metabolic Glycoengineering (MGE), opens up the possibility of altering the cell’s glycocalyx via click chemistry using a two-step approach. This study expands the field of MGE by showing the successful metabolic incorporation of novel alternative artificial glucosamine derivatives. The latter were either deoxygenated or blocked by methyl ether in position 4 to generate deficient glycosylation patterns, while being extended by an alkyne to enable click chemistry as a one-step approach. As a result, we observed a reduced proliferation rate of melanoma cells. Furthermore, using a lectin array, the decrease in high mannose epitopes was observed. In summary, the successful use of alternative artificial glucosamine derivatives enabled a significant alteration in the glycocalyx, consequently influencing cell behavior.

## 1. Introduction

The glycocalyx, a complex and dynamic structure composed of glycoproteins, glycolipids, and proteoglycans, plays a crucial role in cellular biology [1,2,3,4].

Situated on the cell surface, the glycocalyx acts as a multifunctional interface, mediating interactions between cells and their environment [5]. This intricate structure is involved in various cellular processes, including cell adhesion [6,7], signal transduction [8,9], and immune response modulation [10,11]. Understanding the importance of the glycocalyx in these fundamental cellular activities provides key insights into its broader significance within the cell.

One area where the glycocalyx exerts a profound impact is cancer biology, with a particular focus on melanoma, a malignant skin neoplasm known for its aggressiveness [12,13,14] and resistance to conventional therapies [15,16]. The glycocalyx is intimately involved in various stages of melanoma progression, contributing to the intricate network of interactions that govern tumor growth [17], invasion [18], and metastasis [19]. The aberrant glycosylation patterns observed in melanoma cells further underscore the importance of the glycocalyx in shaping the tumor microenvironment. In detail, the glycocalyx plays a crucial role in mediating cell adhesion, facilitating the attachment of melanoma cells to the extracellular matrix and neighboring cells [20]. This promotes migration, a key process in tumor invasion and metastasis. Alterations in glycocalyx composition can enhance the ability of melanoma cells to migrate and invade surrounding tissues [21]. In addition, the glycocalyx is implicated in the metastatic spread of melanoma. Changes in glycan patterns can enhance the ability of melanoma cells to invade blood vessels and disseminate to distant organs [22]. The interaction between the glycocalyx and components of the vascular system plays a critical role in the metastatic cascade, allowing melanoma cells to establish secondary tumors in distant sites [23].

Glycoengineering, a rapidly advancing field, has emerged as a powerful tool for manipulating the glycocalyx. This technique involves the deliberate modification of glycan structures to engineer specific cellular responses. Enzymatic glycan remodeling can involve the use of glycosyltransferases and glycosidases to selectively install or remove specific glycans on proteins, lipids [24], or RNA [25]. This approach allows for site-specific glycan modifications, offering fine control over cellular functions [24,26,27,28].

On the other hand, chemoenzymatic synthesis integrates chemical synthesis and enzymatic transformations to create complex glycan structures with high efficiency and selectivity [29]. Glycoengineering opens up a realm of possibilities, enabling researchers to modulate cell adhesion [30], enhance drug delivery [31], and design targeted therapeutics [32,33].

In this context, we explore an application of glycoengineering by using chemically modified sugars and its transformative potential in shaping cellular behavior [34,35,36].

**Figure 1:**
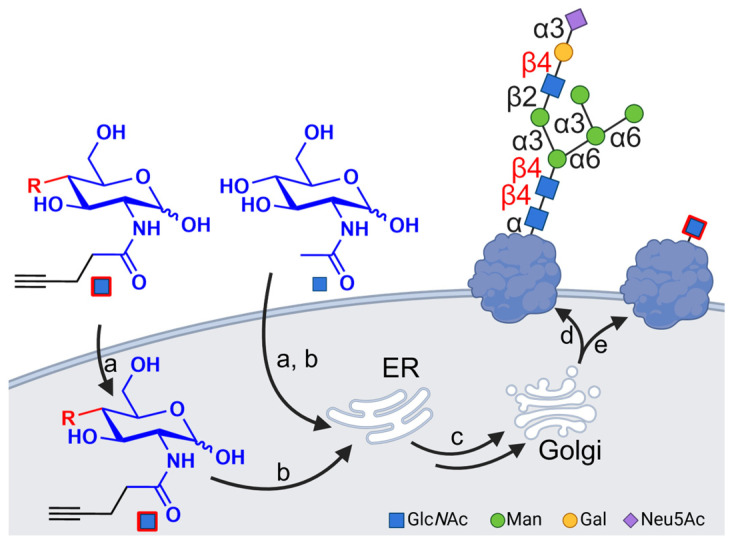
The metabolic pathway of glucosamine derivatives incorporated into *N*-glycan chains of glycoproteins. (a) The uptake of monosaccharides in the cytosol; (b) metabolic activation and incorporation in the core *N*-glycoproteins; (c) transport to the Golgi modifying the glycan chain; (d) an example of natural *N*-glycan located at the cell surface; (e) an example of the use of modified monosaccharide effects’ alteration of cell surface glycoconjugates. Created with BioRender.com.

In this study, we developed artificial glucosamine derivatives to induce changes in the glycocalyx in a highly controlled manner to finally understand the significance of resulting changes in cellular physiology and the role of the glycocalyx in melanoma (Figure 1). We modified the glycocalyx in a controlled manner by newly blocking position 4 of *N*-acetylglucosamine using the methyl ether as a more promising candidate as opposed to the 4-deoxy analog. By exploring the potential applications of glycoengineering and deciphering the molecular mechanisms linking the glycocalyx to melanoma progression, this research contributes to the evolving landscape of cancer biology and opens new avenues for therapeutic interventions.

## 2. Methods

### 2.1. Chemicals and Reagents

The solvents used for purification processes (dichloromethane, cyclohexane, and ethylacetate) were distilled before use. The (Trimethylsilyl)diazomethane solution and d-(+)-glucosamine hydrochloride were purchased from Alfa Aesar (Thermo Fisher Scientific^TM^, Waltham, MA, USA). The remaining chemicals were purchased from Sigma Aldrich (Sigma Aldrich, St. Louis, MO, USA). Perfluorophenyl pent-4-ynoate was synthesized according to the literature [37]. All reactions were performed under a nitrogen atmosphere using anhydrous solvents. Analytical thin-layer chromatography (TLC) was performed on ALUGRAM^®^ Xtra SIL G/UV254 (MACHEREY-NAGEL GmbH & Co., KG, Düren, Germany), aluminum sheets coated with silica gel 60 (layer thickness: 0.20 mm) and fluorescence indicator UV254. The monosaccharides were stained with a sulfuric acid solution (600 mg *N*-(1-Naphthyl)-ethylenediamine, 100 mL sulfuric acid, 2 L MeOH). UV-active compounds were detected with a UV lamp (Vilber, Collégien, France) at 254 nm. The composition of the solvent mixtures is indicated in a volume ratio (*v*:*v*). Nuclear magnetic resonance (NMR) spectra were recorded on a Bruker Avance III HD 400 NMR spectrometer (Bruker Corporation, Billerica, MA, USA) at 25 °C (^1^H: 400 MHz, ^13^C: 100 MHz). Chemical shifts (δ) are given in parts per millions (ppm) and coupling constants (*J*) are given in Hz. Spectra were calibrated concerning the residual solvent signal (δ(CDCl_3_) = 7.26 ppm, δ(CD_3_OD) = 3.31 ppm, and δ((CD_3_)_2_SO) = 2.50 ppm for ^1^H measurements and δ(CDCl_3_) = 77.0 ppm, δ(CD_3_OD) = 49.0 ppm, and δ((CD_3_)_2_SO) = 39.5 ppm for ^13^C measurements). The signal assignment was conducted with additional information of DEPT 135 and 2 D spectra (^1^H, ^1^H)-COSY, (^1^H, ^13^C)-HSQC, and (^1^H, ^13^C)-HMBC. The high-resolution mass spectrometry (HRMS) was performed with a Bruker Daltonics microTOF Focus (Bruker Corporation, Billerica, MA, USA) (electrospray ionization, ESI) (for details, see Supplementary Figure S1).

### 2.2. Cell Lines

Melanoma cell line MelIm (RRID: CVCL_3980) was grown in a DMEM low-glucose medium supplemented with 1% penicillin/streptomycin and 10% FCS (all obtained from Sigma-Aldrich) as described previously [38,39]. Cells were cultured at 37 °C in an atmosphere containing 8% CO_2_. Upon reaching an approximate confluence of 80%, cells were then either subcultured or counted using a Neubauer chamber (Karl Hecht, Sondheim vor der Rhön, Germany). It is essential to note that all experiments were conducted using cells confirmed to be free of mycoplasma contamination, as determined by the mycoplasma detection system MycoSEQ (Thermo Fisher Scientific^TM^).

### 2.3. Analyses of Sugar Incorporation

For evaluating the incorporation of the respective sugars, 4000 cells were seeded onto coverslips in a 12-well plate. The cells were treated with 100 μM or 500 μM of specific sugar, and H_2_O was used as the negative control. Following a 72 h treatment period, the cells underwent a click reaction. For the CuAAC click reaction, a phosphate-buffered saline (PBS) working solution containing 50 μM CuSO_4_ (Carl Roth GmbH + Co., KG, Karlsruhe, Germany), 250 μM THPTA (Carl Roth GmbH + Co. KG), and 2.5 mM sodium ascorbate (Sigma Aldrich) was prepared and reacted at room temperature for one hour. For the CuAAC click reaction, 20 μM Cy5-azide was freshly added to the working solution. The reaction process involved removing the medium, washing the cells once with PBS, and subsequently incubating them for 5 min at 37 °C and 8% CO_2_. Afterwards, the cells were washed three times with PBS and allowed to recover in a fresh growth medium for 2 h. Fixation was performed by removing the medium, washing once with PBS, and fixing the cells for 15 min with a 4% paraformaldehyde solution in PBS at room temperature. For the cell nucleus staining, a DAPI solution (1:10,000 in PBS, Sigma Aldrich) was used and the cells were mounted on a microscopic slide by using Aqua-Poly/Mount (Polysciences, Warrington, PA, USA). The analysis of the final staining was conducted by using an Olympus IX83 inverted microscope (Olympus, Tokyo, Japan) paired with Olympus CellSens Dimension software (Version 2.3, Olympus). Representative images were adjusted uniformly for brightness and contrast to enhance staining visibility.

### 2.4. Degradation of Sugars in Glycocalyx

To assess the stability of sugars within the glycocalyx, 4000 cells were seeded onto microscope coverslips within a 12-well plate. The cells were treated with 500 μM Glc*N*Al **1**, while H_2_O served as the negative control. Following a 72 h treatment duration, the cell culture medium was replaced and renewed. Subsequently, the click reaction and cell fixation were performed as described in the previous section, and were carried out on days 0, 1, 2, and 3.

### 2.5. Protein Extraction

A total of 75,000 cells were seeded per well onto a 6-well plate for the protein analysis. These cells were treated with either 100 μM or 500 μM of a particular sugar, while H_2_O served as the negative control. Cells were also treated with 10 μM or 20 μM NGI-1 (inhibitor of *N*-glycosylation, SML1620, Sigma-Aldrich) and a control (DMSO). After 72 h of treatment, cells were harvested, resulting in cell pellets. These pellets were lysed in 100 μL of an RIPA buffer (Thermo Fisher Scientific^TM^) for 20 min at 4 °C. Centrifugation at 13,000 rpm for 10 min at 4 °C was performed to remove cell fragments, and the resulting supernatant was collected. The protein content was determined by the BCA kit and carried out according to the manufacturer’s instructions (Pierce^TM^ BCA Protein Assay Kits, Thermo Fisher Scientific^TM^) like in previous protocols [40].

### 2.6. Western Blot

Western blots were performed as described previously [41]. In total, 40 μg of protein lysates was loaded onto a 10% SDS polyacrylamide gel for electrophoresis. After electrophoresis, the proteins were blotted onto a PVDF membrane (Bio-Rad, Hercules, CA, USA) and the membrane was blocked with 5% bovine serum albumin (BSA)/Tris-buffered saline–Tween 20 (TBS-T) for 1 h. The primary antibody targeting SLC38A1 (SNAT1) (1:5000 in 5% BSA/TBS-T, cat. 36,057, Cell Signaling Technology, Danvers, MA, USA) was incubated overnight by 4 °C. The primary antibody against β-actin (1:3000 in TBS-T, A5441, Sigma Aldrich) was incubated for 1 h at room temperature. The corresponding secondary antibodies were HRP-conjugated anti-rabbit and anti-mouse antibodies (1:2000 in TBS-T, cat. 7074 and 7076, Cell Signaling), also being used for 1 h. Protein detection was achieved by adding a Clarity^TM^ Western ECL Substrate (Bio-Rad) and visualized using a Chemostar chemiluminescence imager (Intas, Goettingen, Germany). The quantitative analysis of signal intensity was carried out by using LabImage software (Version 4.2.3, Kapelan Bio-Imaging GmbH, Leipzig, Germany).

### 2.7. Precipitation of Glycoproteins

Following the Vector Laboratories Inc. (Marek, CA, USA) protocol, 50 μL of the extracted protein solution (1–5 mg/mL) was labeled with the biotin-azide reagent (Azide-PEG3-biotin conjugate, Sigma-Aldrich). The reaction mixture, containing 100 mM THPTA, 20 mM CuSO4, 300 mM sodium ascorbate, and 25 μM biotin-azide, was supplemented with PBS to 200 μL. The click reaction was performed for one hour at room temperature, including rotation on a Bio-Rad PowerPac (Bio-Rad Laboratories, Inc., Hercules, CA, USA) while being shielded from light. The magnetic streptavidin beads (Streptavidin Mag SepharoseTM, Cytiva, Marlborough, MA, USA) were used to isolate the biotinylated proteins as described with a modification of the manufacturer’s protocol. Initially, 100 μL of beads was added, followed by supernatant removal. The beads were resuspended in TBS and the discharge was removed. A mixture of 100 μL PBS and the clicked protein solution was combined with the beads and incubated overnight at 4 °C with orbital shaking. Beads were washed three times with 2 M UREA/TBS (pH 7.5). Afterwards, 50 μL of a 2% SDS solution in PBS dissolved the proteins by 95 °C for a span of minutes. The supernatant, containing dissolved proteins, was transferred to a new reaction tube, followed by the addition of 10 μL PBS for protein stabilization.

### 2.8. Silver Staining

The silver staining procedure followed the instructions provided by the manufacturer (Invitrogen^TM^ SilverQuest^TM^, Thermo Fisher Scientific^TM^) and was conducted on a 10% SDS gel [42]. A total of 30 μL of the precipitated glycoproteins was loaded onto the gel for the analysis.

### 2.9. Precipitation Western Blot

The Western blot analysis was adapted for the analysis of precipitated glycoproteins. A 10% SDS gel was loaded with 30 μL of the glycoprecipitate combined with a Laemmli sample buffer (ROTI^®^Load, Carl Roth GmbH + Co. KG, Nürnberg, Germany) and electrophoresis and blotting were performed as described in the previous section. The membrane underwent blocking by using 5% BSA/TBS-T at 4 °C overnight. Subsequently, the blot was exposed to Poly-HRP Streptavidin (diluted at 1:6000 in TBS-T, Pierce^TM^ Streptavidin Poly-HRP, Thermo Fisher Scientific^TM^) and incubated for one hour at room temperature. Following three washing steps with TBS-T, the analysis was performed as previously described.

### 2.10. Proliferation Assays

To assess proliferation, 5000 cells were seeded per well into a 12-well plate. The culture medium was supplemented directly with either sugars at concentrations of 100 μM or 500 μM, or with a control (H_2_O). Following incubation for 24, 48, and 72 h, cells from each treatment group were detached using trypsin and subsequently counted using a Neubauer chamber to determine cell numbers.

### 2.11. XTT Assay

Cell proliferation and mitochondrial activity were determined by utilizing Cell Proliferation Kit II (XTT) (Roche Diagnostics GmbH, Mannheim, Germany). A total of 1000 cells were seeded per well in a 96-well plate, and the XTT medium was supplemented directly with concentrations of 500 μM and 100 μM of the corresponding sugar, or with the control (H_2_O). Cells were also treated with 10 μM or 20 μM NGI-1 and a control (DMSO). Following the manufacturer’s protocol and previous works [43], the XTT reagent was introduced to the adherent cells at 24, 48, and 72 h intervals post-seeding. The absorbance was measured at 490 nm using a CLARIOstar plate reader (BMG Labtech GmbH, Ortenberg, Germany).

### 2.12. Clonogenic Assay

To investigate attachment-dependent colony formation and growth of cancer cells, clonogenic assays were conducted following previously established protocols [41,44]. For this assay, either 100 or 200 cells were seeded into individual wells within 6-well plates. Subsequently, the cells were exposed to concentrations of 100 μM or 500 μM of the specified sugars and the control (H_2_O). Also, cells were treated with NGI-1 (10 μM and 20 μM) and a control (DMSO). Following a 7-day incubation period, the cells were fixed on the plates, and both the number and size of the colonies were quantified and recorded.

### 2.13. Lectin Assay

Cells were treated with 500 μM Glc*N*Al **1** or 4-*O*Me-Glc*N*Al **2** in PBS for 72 h. The cells were harvested as described in previous sections. The glycocalyx changes were measured using the Cells LEctPROFILE^®^ kit (LK04CellsE, GLYcoDiag, Orléans, France). The manufacturer’s protocol was followed [45]. Following the washing and centrifugation steps, the cells were resuspended in PBS treated with carboxyfluorescein diacetate succinimidylester (CFDA-SE, Sigma-Aldrich) to label the cells. Thereafter, approximately 200,000 labeled cells suspended in 100 μL PBS were added to each well of the plates. The plates were incubated protected by light at room temperature with gentle agitation for 2 h. After washing with PBS, the fluorescence intensity was quantified using a CLARIOstar plate reader and using excitation as follows: wavelength = 485 nm and emission wavelength = 530 nm. (Abbreviations used: Concanavalin-A (ConA), *Pisum sativum* agglutinin (PSA), *Galanthus nivalis* lectin (GNA), *Hippeastrum hybrid* lectin (HHA), *Amaranthus caudatus* (ACA), *Bauhinia purpurea* lectin (BPA), *Dolichos biflorus* (DBA), *Helix pomatia* agglutinin (HPA), *Wisteria floribunda* (WFA), *Maclura pomifera* (MPA), *Artocarpus integrifolia* (AIA), *Agaricus bisporus* (ABA), Peanut (*Arachis hypogaea*) (PNA), *Datura stramonium* (DSA), *Griffonia simplicifolia*-II (GSL-II), Wheat germ agglutinin (WGA), *Lotus tetragonolobus* lectin (LTA), *Ulex europaeus* agglutinin-I (UEA-I), *Maackia amurensis* (MAA), *Sambucus nigra* (SNA), *Phaseolus vulgaris*-E (PHA-E), and *Phaseolus vulgaris*-L (PHA-L)).

### 2.14. Statistical Analysis

The statistical analysis was conducted by using GraphPad Prism software (Version 10.10). The same software was utilized to generate the graphical representations. The results are expressed as the mean ± Standard Error of the Mean. Student’s *t*-test or a One-Way ANOVA with a multiple comparison test (Dunett) was employed to assess differences between groups, with statistical significance denoted by a *p*-value less than 0.05 (* for *p* < 0.05, ** for *p* < 0.005, and *** for *p* < 0.001), while outcomes that were not statistically significant (*p* > 0.05) were not labeled.

## 3. Results

### 3.1. Synthesis of Artificial Glucosamine Derivatives

**Figure 2:**
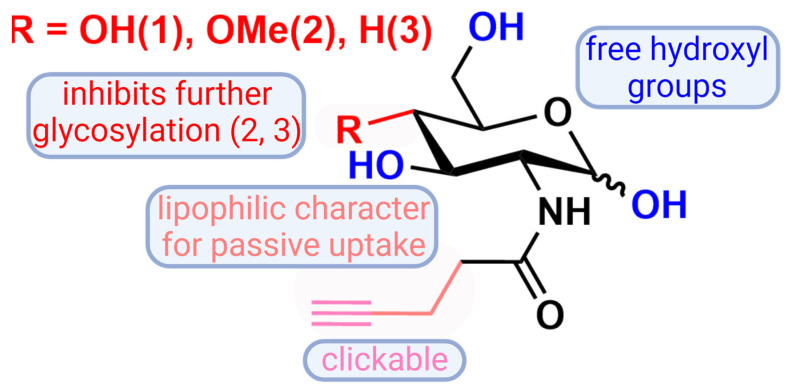
A graphical representation of 2-*N*-(4′-pentynoyl)-d-glucopyranosylamine (Glc*N*Al) **1** and the novel glucosamine derivatives 4-Methoxy-2-*N*-(4′-pentynoyl)-d-glucopyranosylamine **2** and 4-deoxy-2-*N*-(4′-pentynoyl)-d-glucopyranosylamine **3** with reference to their properties. Created with BioRender.com.

In this study, we anticipate that the metabolic incorporation of modified *N*-Acetylglucosamine (Glc*N*Ac) directly influences the glycan composition. Hence, the altered sugar backbone leads to the inhibition of 1–4 glycosidic linkages. Moreover, the natural moiety is expanded by an alkyne carrying acyl chain (Figure 2).

To address the issue of the apolar *C*-4 moiety of our artificial sugars and their comparably long-range alkyne tagged acyl chain, we decided to use monosaccharides that carry free hydroxyl groups. Thus, the partly acetylated compounds are excluded to allow for deficient *N*-glycan chains and to prevent nonspecific cysteine *S*-glycosylation induced by per-O-acetylated unnatural monosaccharides during metabolic glycan labeling, which was observed by Qin et al. [46]. Therefore, we synthesized *C*-4 modified glucosamine derivatives **2** and **3** and characterized the three glucosamine derivatives carrying a biorthogonal clickable group (Glc*N*Al **1**, 4-*O*Me-Glc*N*Al **2**, and 4-deoxy-Glc*N*Al **3**, Figure 2). Two of them were modified at *C*-4, to block the elongation of the glycan chains.

**Scheme 1:**
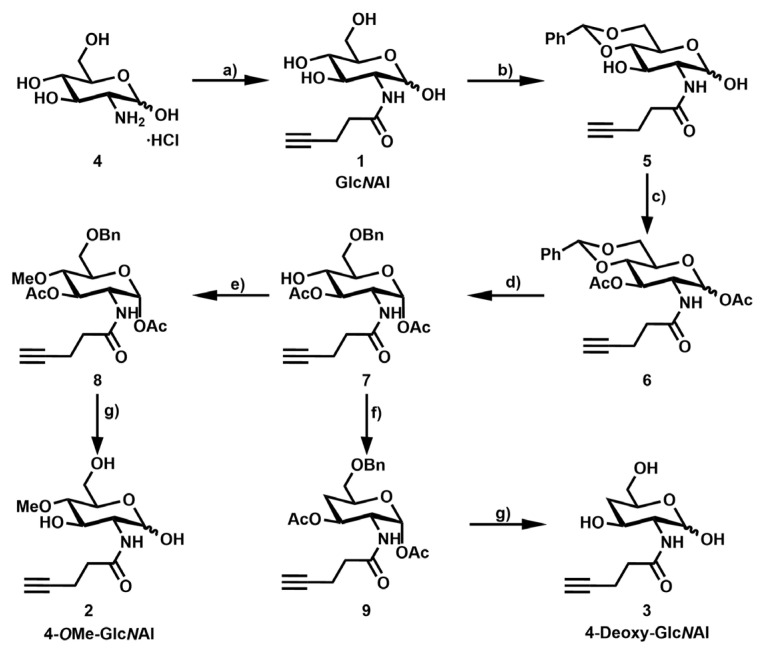
Synthesis of 4-*O*Me-Glc*N*Al **2** and 4-deoxy-Glc*N*Al **3**: (a) Perfluorophenyl pent-4-ynoate, diisopropylethylamine, dimethylformamide, rt, 18 h, 96%; (b) PhCH(OMe)_2_, camphorsulfonic acid, acetonitrile, rt, 3 d, 79%; (c) acetanhydride, pyridine, rt, 18 h, 99%; (d) trifluoroacetic acid, trifluoroacetic anhydride, Et_3_SiH, dichloromethane, 0 °C → rt, 4 h, 79%; (e) Me_3_SiCHN_2_, HBF_4_, dichloromethane, 0 °C → rt, 18 h, 43%; (f) (i) Thiocarbonyldiimidazole, toluene, 110 °C, 2.5 h, and (ii) Bu_3_SnH, azobisisobutyronitrile, 110 °C, 30 min, 54%; (g) (i) 2,3-dichloro-5,6-dicyano-1,4-benzoquinone, dichloromethane, 45 °C, 1 d, 62%, and (ii) NaOMe, MeOH, rt, 2 h, 50–72%.

Scheme 1 depicts the synthesis for the three glucosamine derivates: Following a standard procedure, commercially available glucosamine hydrochloride 4 and perfluorophenyl pent-4-ynoate were conjugated under basic conditions to yield the amide Glc*N*Al **1** in 96%. In the following steps, an acetal was formed with benzaldehyde, allowing the protection of the two hydroxyl groups at *C*-4 and *C*-6a and the remaining hydroxyl groups were acetylated with Ac_2_O in pyridine to form the fully protected derivative **5**. Under reducing conditions, the introduced acetal was reacted with TFA, TFAA, and Et_3_SiH to selectively synthesize the 6-benzyl ether **7** in good yields. At this point, the linear synthesis was split to produce a methyl ether at *C*-4 on the one hand. Because of the alkaline labile protecting groups, compound **7** was reacted with (Trimethylsilyl) diazomethane under acidic conditions using HBF_4_. Glucosamine derivative **7** was deoxygenated at *C*-4. Therefore, the reaction was performed under Barton–McCombie conditions. The reduced compound was isolated after 3 h at 110 °C. Afterward, the protecting groups of both compounds (**8** and **9**) were removed. First, the benzyl ether was oxidized overnight under comparably mild conditions using DDQ. Secondly, the esters were hydrolyzed following a standard procedure with NaOMe in methanol. Target compounds as well as intermediates were characterized by ^1^H and ^13^C NMR spectroscopy and verified by mass spectrometry (see Supplementary Materials).

### 3.2. Incorporation of Modified N-Acetylglucosamines in Glycocalyx

**Figure 3:**
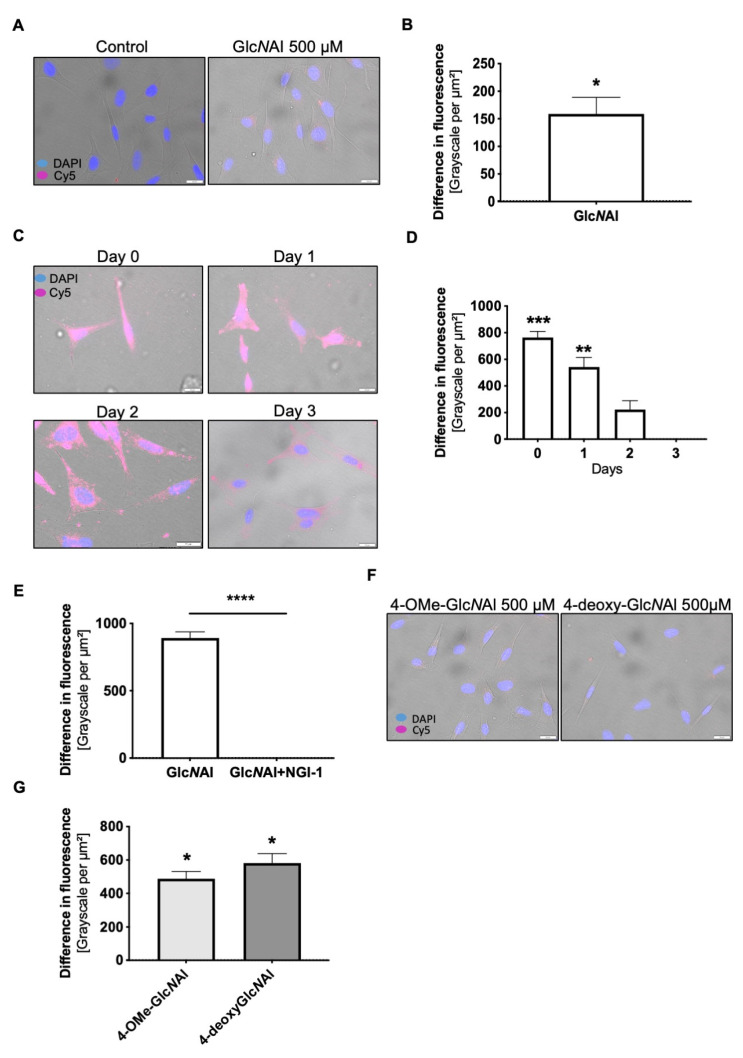
(**A**) Fluorescence images of the cell line MelIm treated with Glc*N*Al **1** at 500 µM or the control (H_2_O), respectively, with (**B**) showing the differences between the control and treatment. (**C**) Exemplary fluorescence images showing the temporal course of the decline in glycocalyx modification from day 0 to day 3 after treatment with Glc*N*Al **1** or the control (H_2_O) and (**D**) presenting the differences between the control and treatment from day 0 to day 3. (**E**) The differences in the fluorescence signal between cells treated with 500 µM Glc*N*Al **1** or a combination of 20 µM NGI-1 and 500 µM Glc*N*Al **1**. (**F**) Exemplary fluorescence images of 4-*O*Me-Glc*N*Al **2**- and 4-deoxy-Glc*N*Al **3**-treated cells, with (**G**) depicting the differences in fluorescence between control and treated cells. (**B**,**D**,**F**,**G**) Differences in the fluorescence signal are presented as a grayscale per µm^2^ for the Cy5 fluorescence signal and were measured from eleven random cells. For (**A**–**G**), cells were pre-treated with 500 µM of the respective sugars (Glc*N*Al **1**, 4-*O*Me-Glc*N*Al **2**, or 4-deoxy-Glc*N*Al **3**) or the respective controls (H_2_O) for 72 h. The click reaction was performed by incubating the cells with 50 µM CuSO_4_, 250 µM THPTA, 2.5 mM sodium ascorbate, and 20 µM Cy5-azide for 5 min. Cells were further cultivated for 2 h in a growth medium and eventually fixed with 4% paraformaldehyde and further processed for staining as described in the Methods Section. All data from at least three independent experiments are represented as mean ± SEM (*: *p* <  0.05, **: *p* <  0.01, ***: *p* <  0.001, ****: *p* < 0.0001). Scale bars = 20 µm.

Firstly, the clickable derivative of *N*-Acetylglucosamine (Glc*N*Al **1**, Figure 2) was introduced to explore its potential incorporation into and retention within the glycocalyx. Glc*N*Al **1** was employed as a control substance with the expectation that it would not have a significant influence on the glycocalyx. Subsequent experiments were conducted using two different concentrations of Glc*N*Al **1** (100 and 500 µM, data for 100 µM are not shown). The assessment involved the covalent attachment of a visibly fluorescent dye (Cy5-Azid) through the Copper-Catalyzed Azide–Alkyne Cycloaddition (CuAAC) [47,48] click reaction. As depicted in Figure 3A, the successful incorporation of sugars onto the cell surface was observed by a markedly higher fluorescent signal compared to the control-treated cells (Figure 3B). The integration of sugars into the glycocalyx was rapid, depicting a strong signal already after 24 h. Over the initial two days, a consistent signal was observed. However, a notable decline in the signal was recorded on day 3 (Figure 3C,D). To ensure that our modified sugars are incorporated into the glycocalyx, we treated cells with Glc*N*Al **1** together with the *N*-glycosylation inhibitor NGI-1. The significant reduction in signal intensity, which results from NGI-1 treatment compared to cells treated with Glc*N*Al **1** alone, supports that the modified sugar is being incorporated into the glycocalyx (Figure 3E).

After the successful evaluation of Glc*N*Al **1**, two modified Glc*N*Ac variants were examined: 4-*O*Me-Glc*N*Al **2** and 4-deoxy-Glc*N*Al **3**. As illustrated before, the incorporation of the two new sugars into the glycocalyx was assessed. The click reaction was performed, mirroring the methodology used for Glc*N*Al **1**. Both artificial monosaccharides were incorporated into the glycocalyx (Figure 3F) and the measurement of the grayscale per µM² showed a significant induction compared to the control in the form of GlcNA1 **1** (Figure 3G, data for 100 µM are not shown).

### 3.3. Changes in Protein Glycosylation

Our initial objective was to provide qualitative evidence for changes in protein glycosylation patterns induced by modified sugars. Beyond general fluorescent labeling of the cell surface, we aimed to examine the potential impact of these sugars on protein post-translational modifications.

**Figure 4:**
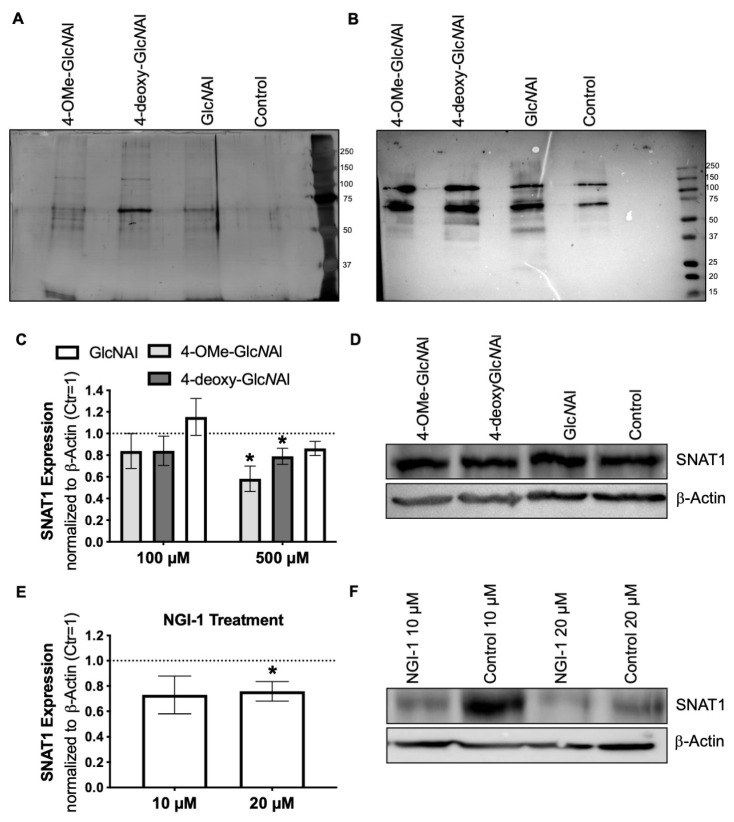
(**A**) Exemplary silver gel staining depicting the precipitation of glycoproteins into which the clickable sugars were incorporated. MeIIm cells were treated with 500 µM of the specific sugars, alongside a control group (H_2_O). (**B**) The Western blot analysis illustrating the precipitation of glycoproteins facilitated by the employment of Streptavidin Poly-HRP. (**C**) Densitometric measurement of SNAT1 expression after treatment with 100 µM (*n* = 6) and 500 µM (*n* = 4) of the corresponding sugars. (**D**) An exemplary Western blot image of the treatment with 500 µM of the corresponding sugars. (**E**) Densitometric measurement of SNAT1 following treatment with 20 and 50 µM NGI-1 or the respective control (DMSO) (*n* = 5). (**F**) An exemplary Western blot image of SNAT1 expression after the treatment with NGI-1. All data from at least three independent experiments are represented as the mean ± SEM (*: *p* <  0.05).

As previously mentioned, cells were treated with 100 µM or 500 µM of each sugar for 72 h and subsequently lysed. *N*-glycoproteins present in the lysate were labeled using the copper(I)-catalyzed azide alkyne cycloaddition (CuAAC) click reaction with a biotin-azide reagent. Labeled proteins were precipitated using magnetic streptavidin beads. The obtained precipitate was subjected to SDS-PAGE and initially examined via silver staining, revealing differences in banding patterns, particularly in the region between 50 kDa and 75 kDa (Figure 4A). To gain further insights, the lysate was applied to SDS-PAGE and subsequently blotted. Detection using Streptavidin Poly-HRP is a highly sensitive method, capable of identifying even faint bands. When comparing Glc*N*Al **1** to the other two sugars, blocked for further glycosylation in position 4, with regard to changes in molecular weights of glycosylated proteins, in general, in the range of 50 kDa to 100 kDa, the most pronounced changes were observed (Figure 4B, left). A treatment of 100 µM is not presented, as it did not lead to visible changes. Due to the qualitative changes in protein glycosylation described previously, we decided to focus on a model protein for a more detailed analysis.

Our choice fell on the amino acid transporter SLC38A1 (SNAT1), which is known for its multiple *N*-glycosylation sites.

Here, cells were treated as previously described with the sugars (100 µM and 500 µM) or a control (H_2_O) for 72 h and lysed. Additionally, cells were treated with NGI-1 (10 µM and 20 µM) along with the control (DMSO). Changes in SNAT1 were investigated using an antibody specific to SNAT1 in the Western blot analysis. Treatment with 4-*O*Me-Glc*N*Al **2** and 4-deoxy-Glc*N*Al **3** (500 µM) resulted in a significant reduction in band intensity (Figure 4C). This reduction is prominently visible upon comparison (Figure 4D). The unmodified sugar Glc*N*Al **1** did not result in significant changes as anticipated. No significant differences were observed using the concentration of 100 µM. We focused on identifying the optimal dose of altered Glc*N*Al **1** to achieve maximum effects, as higher concentrations may lead to decreased SNAT1 expression. Furthermore, as anticipated, treatment with NGI-1 led to a notable decrease (Figure 4F) in band intensity, particularly at a concentration of 20 µM (Figure 4E). The fact that both, the inhibitor NGI-1 and the modified sugars, lead to a significant reduction in band intensity strengthens the assumption that the sugars reduce *N*-glycosylation in proteins.

### 3.4. Effects on Cellular Level

**Figure 5:**
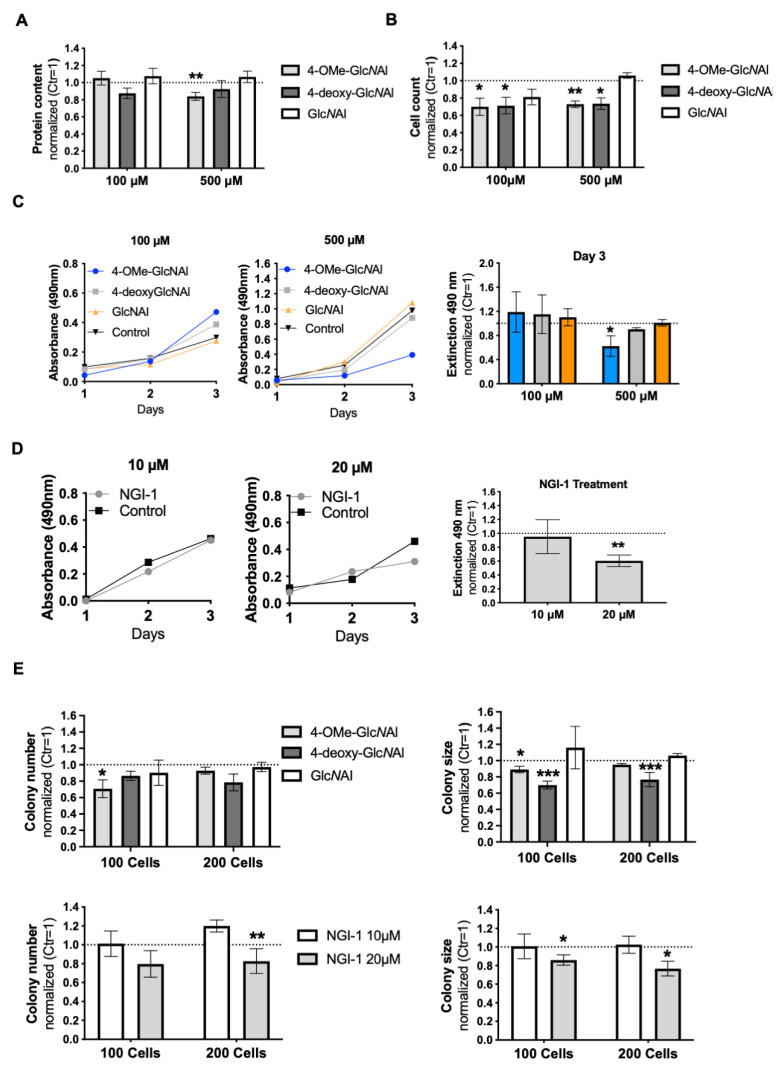
(**A**) The normalized protein concentration of MelIm cells after treatment with 100 µM (*n* = 8) and 500 µM (*n* = 4) of the respective sugar. (**B**) Measurement of proliferation by cell counting using the Neubauer chamber (each *n* = 3). (**C**) An exemplary time course of XTT absorbance curves representing treatments with 100 µM and 500 µM concentrations of the respective sugars and control (H_2_O) (left panel). Absorbance on day 3 is presented (right panel). (**D**) An exemplary time course of XTT absorbance curves displaying the treatment with NGI-1 at 10µM and 20 µM and control (DMSO) (left panel). Absorbance on day 3 is presented (right panel). (**E**) The normalized colony number and colony size of a clonogenic assay of MelIm cells treated with 500 µM of the respective sugars (upper panels) and 10 µM and 20 µM of the inhibitor NGI-1 (lower panels). Each treatment was replicated four times. All data from at least three independent experiments are represented as the mean ± SEM (*: *p* <  0.05, **: *p* <  0.01, ***: *p* <  0.001).

We further aimed to demonstrate that the observed changes might indicate a change in cell proliferation due to possibly decreased *N*-glycosylation. Treatment of cells with 4-*O*Me-Glc*N*Al **2** at a concentration of 500 µM revealed a significant reduction in total protein content (Figure 5A). However, at a lower concentration (100 µM), this decrease was not statistically significant. For 4-deoxy-Glc*N*Al **3**, a tendency toward decreased protein content was observed at both concentrations, although statistical significance was not reached. As anticipated, Glc*N*Al **1** did not lead to any significant differences from the control group. Given the reduction in total protein content, we inferred that these effects might be due to inhibited proliferation. To investigate this, cell proliferation was assessed by treating cells with 100 µM and 500 µM of the modified sugars. Significant reductions in proliferation were observed for 4-*O*Me-Glc*N*Al **2** and 4-deoxy-Glc*N*Al **3** compared to the control after 72 h (Figure 5B). The control sugar, Glc*N*Al **1**, had no significant effect on proliferation. For a more sensitive assessment, an additional XTT assay was conducted. Over 3 days, only treatment with 500 µM 4-*O*Me-Glc*N*Al **2** led to a significant reduction in proliferation (Figure 5C), while Glc*N*Al **1** showed no effect. To confirm these results, NGI-1 was used as a reference in the XTT assay. A concentration of 20 µM NGI-1 resulted in a significant reduction in proliferation, similar to 4-*O*Me-Glc*N*Al **2** (Figure 5D). These results indicate that both 4-*O*Me-Glc*N*Al **2** and the *N*-glycosylation inhibitor NGI-1 lead to decreased proliferation.

To validate these findings, we examined the influence of the sugars on colony formation. Individual MelIm cells were seeded and were treated with the corresponding sugars (100 µM and 500 µM) and NGI-1 (10 µM and 20 µM) for one week. Both NGI-1 (20 µM) and 4-deoxy-Glc*N*Al **3** have a strong negative influence on the colony size. Furthermore, 4-*O*Me-Glc*N*Al **2** also showed a tendency to reduce the colony size. In addition, this sugar was able to significantly reduce the number of colonies. Glc*N*Al **1** neither had an influence on the size nor the number colony (Figure 5E). Colony formation assays are shown in the Supplement (Supplementary Figure S2). The fact that similar outcomes were observed for both, NGI-1 and the sugars, further supports our findings.

### 3.5. Assessment of Defined Changes in Glycocalyx Using Modified GlcNAc

**Figure 6:**
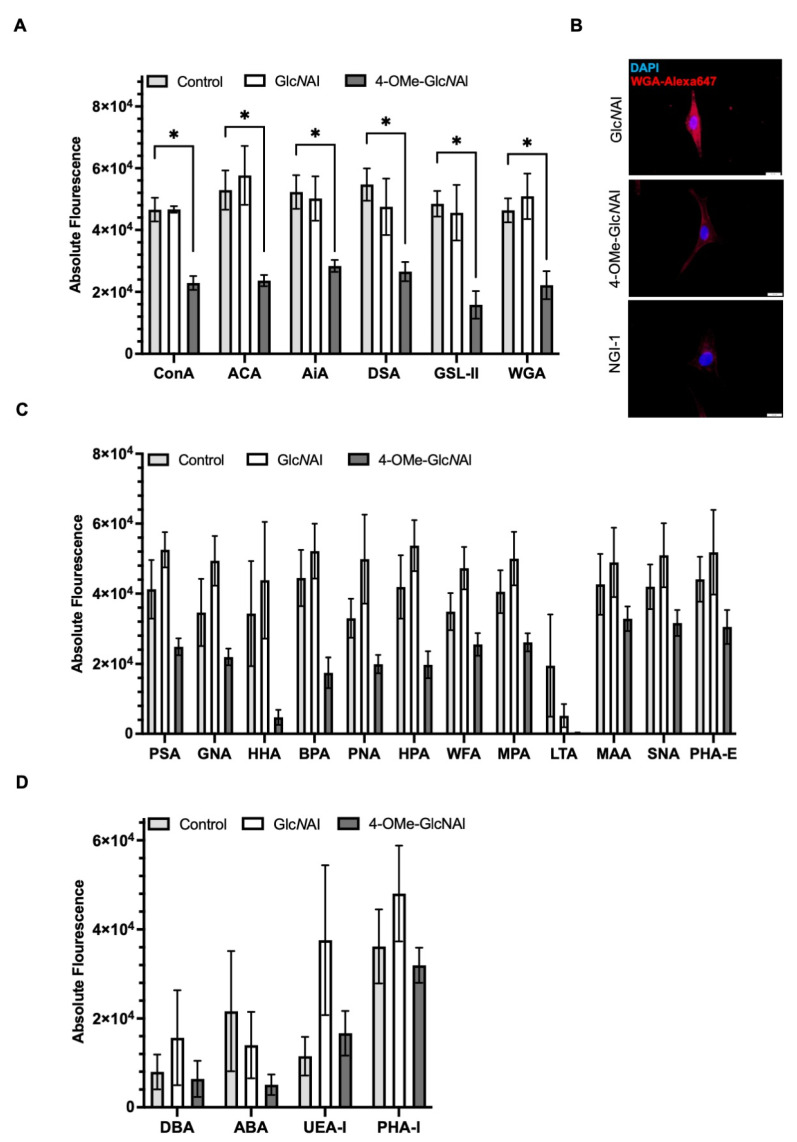
Alterations in surface glycosylation after a 72 h treatment with 500 µM of the sugars Glc*N*Al **1** and 4-*O*Me-Glc*N*Al **2**, and a control (H_2_O). (**A**,**C**,**D**) The lectin assay quantifying the absolute fluorescence intensity of cells bound to different lectins. (**B**) WGA staining of MelIm treated with 500 µM Glc*N*Al and 4-*O*Me-Glc*N*Al, respectively, or 20 µM of NGI-1. Scale bars = 20 µm. Abbreviations are listed in the Methods Section. All data from at least three independent experiments are represented as the mean ± SEM (*: *p* <  0.05).

To elucidate the effects of modified glucosamine derivatives on glycocalyx chains, lectin arrays were employed to analyze changes in specific sugar chain quantities. Therefore, we performed feeding experiments with Glc*N*Al **1** and 4-*O*Me-Glc*N*Al **2** to compare the lectin arrays with untreated cells. After a treatment period of 72 h with the respective sugars, the cells were harvested and labeled. Subsequently, equal amounts of cells were plated into the assay and analyzed. The results of the lectin array can be summarized in three groups:

(a) In the first group, 4-*O*Me-Glc*N*Al **2** resulted in a significant reduction in the cellular binding affinity to certain lectins. These include ConA, ACA, AiA, DSA, GSL-II, and WGA. Conversely, Glc*N*Al **1** demonstrated no effect on the binding affinity of these sugars compared to the untreated control (Figure 6A). Performing staining with fluorescence-labeled WGA, which specifically binds to *N*-acetyl-d-glucosamine, revealed a strong immunofluorescence signal in control-treated cells (Glc*N*Al 1). Cells treated with 4-*O*Me-Glc*N*Al 2 and NGI-1 revealed less to almost no staining, again proving changes in glycocalyx composition after the incorporation of 4-*O*Me-Glc*N*Al (Figure 6B).
(b) In the second group, we observed a similar reduction concerning the binding affinity of 4-*O*Me-Glc*N*Al **2** compared to Glc*N*Al **1** and the control for lectins such as PSA, PNA, GNA, HHA, BPA, HPA, WFA, MPA, MAA, SNA, and PHA-E; however, based on the variances, this did not reach statistical significance (Figure 6C).
(c) In a third group with DBA, ABA, UEA-1, and PHA-L, no influence of the two unnatural monosaccharides on the binding affinity was determined (Figure 6D).

## 4. Discussion

Addressing the glycocalyx to understand its impact on tumor development and progression is an important research area, as the glycan chains are the first interaction with the environment. In cell biology, studies addressing the glycocalyx commonly use broad inhibitors like NGI-1, an inhibitor of oligosaccharyltransferase, or generally digest the glycocalyx. However, these methods are not highly controllable and not specific for defined changes in the glycocalyx. Also in previous studies, fully acylated 4-deoxy-Glc*N*Ac has been demonstrated to inhibit heparan sulfate expression up to ~96% (IC50 = 16 μM) [49,50].

However, in this study, we newly developed structurally and functionally different analogs to overcome these limitations and potentially extend their function of incorporation to biorthogonal conjugation with fluorescent dyes.

We elucidated the impact of artificial glucosamine derivatives that directly interrupt the metabolic elongation of the glycan chains. These are equipped with an alkyne expanded acyl chain that can be used as a visualization probe. The successful incorporation of a similar photocrosslinking Glc*N*Ac analog was reported by Wu et al. [51]. Thereby, proteomics indicates a broad spectrum of different *N*-glycans carrying the unnatural Glc*N*Daz moiety, especially attached to the α1-6Man in the *N*-glycan core [51]. In addition, the C-4 modification in 4-deoxy-Glc*N*Al **3** and 4-*O*Me-Glc*N*Al **2** leads to the disruption of chain elongation by a 1-4 glycosidic linkage. Modifying the glycocalyx in a controlled manner by blocking position 4 of *N*-acetylglucosamine, using the methyl ether as a more promising candidate as opposed to the 4-deoxy analog, is, to our knowledge, new.

This modification should not result in strong changes in *O*-glycosylation by Glc*N*Ac, a ubiquitous post-translational modification of intracellular proteins, as the *C*-4 is not involved in this process. A study of Ma et al. [52] supported this by showing that UDP-4-deoxy-Glc*N*Ac is a substrate for *O*-linked β-*N*-acetylglucosaminyl transferase (OGT), although the affinity to sOGT is slightly reduced.

Labeling the treated cells using click chemistry with a cyanine-5 fluorophore confirmed the incorporation of all three synthetic sugars into the glycocalyx. Since we performed the click reaction on living cells, which have not been fixed or permeabilized before the click reaction, only sugars incorporated into the glycocalyx were labeled with the fluorescent dye Cy5.

Interestingly, the modifications were stable for at least three days, which hints to a turnover rate of approximately this period and suggests a dynamic turnover of glycan structures within this complex environment. Previous studies stated a turnover rate of the glycocalyx between 24 h and 7 days, depending on the cell type and potential exposition to, e.g., growth factors [53]. Since the glycocalyx consists of multiple glycosylated molecules, each displaying its own half-life, the techniques using clickable sugars can only give an estimate over the total glycocalyx. However, general changes in turnover can be quantitatively analyzed using the methods displayed in this study. In addition, using the modified sugar presented in this study, disrupting specific chain elongation is feasible and will give additional information.

To provide a more comprehensive understanding of the anticipated changes in the glycosylation pattern and to induce defined changes in the glycocalyx, we evaluated the impact of 4-*O*Me-Glc*N*Al **2** or 4-deoxy-Glc*N*Al **3** and revealed a general reduction in protein glycosylation, which was confirmed on individual proteins like the amino acid transporter SNAT1. The changes observed are similar to the effects of the general inhibitor NGI-1. Contrary to NGI-1, 4-*O*Me-Glc*N*Al **2** or 4-deoxy-Glc*N*Al **3** have a defined and more controllable effect on specific *N*-glycosylation structures. In contrast, high concentrations of Ac_4_Man*N*Az potentially lead to *S*-glyco-modification side reactions between peracetylated monosaccharides and protein cysteine residues, the induction of a nonspecific labeling of proteins that cannot occur with non-peracetylated analogs [46,54,55].

**Figure 7:**
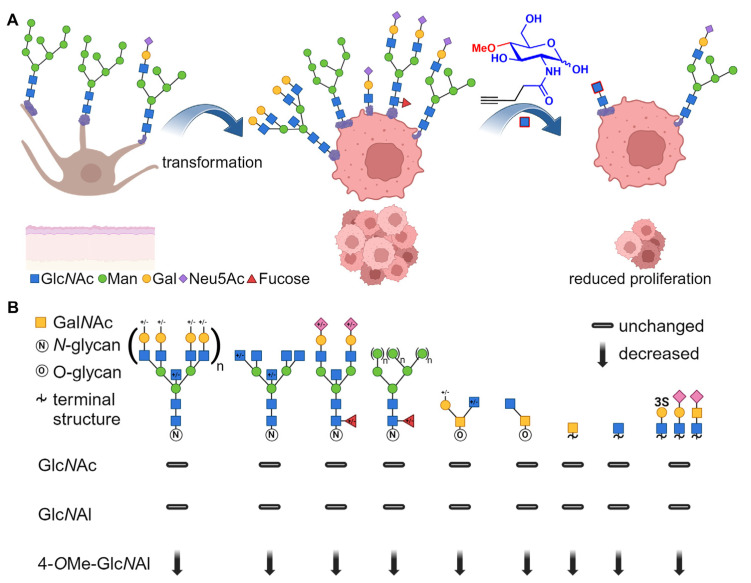
(**A**) A schematic illustration of the glycosylation pattern of normal melanocytes (left) differing from melanoma cells after transformation (middle). By the incorporation of 4-*O*Me-Glc*N*Al **2**, the glycocalyx of the melanoma cells was modified, resulting in reduced proliferation. (**B**) The recognition of carbohydrate epitopes based on a lectin array (signal is relative to negative control) [61]. Created with BioRender.com.

On the cellular level, modifications of the glycosylation pattern with 4-*O*Me-Glc*N*Al **2** and 4-deoxy-Glc*N*Al **3** led to a significant reduction in proliferation. Those results are supported by the fact that both the amount and size of colonies in a clonogenic assay were reduced by the *C*-4 modification of glucosamine derivatives. It is known that changes in *N*-glycosylation can intricately modulate the activity and signaling pathways of growth factor receptors, thus influencing proliferative signaling [56,57,58]. The inhibition of *N*-glycosylation using NGI-1 has been demonstrated to hinder the proliferation of lung cancer, multiple myeloma, or glioma cells [59]. Our newly developed modified sugars resulted in similar effects on melanoma cells. Another attempt to modify *N*-glycosylation is in situ glycan editing to modify the glycocalyx [60]. This enzymatic method of adding, e.g., fucose or sialic acid sugars, can help to analyze specific changes. Yet, it does not use endogenous glycosylation synthesis to modify the glycocalyx, being partially artificial. To summarize, the newly designed Glc*N*Ac variants modify the glycocalyx more specifically. The inhibitory activity can be linked to the defined modification of sugar chains depending on 1-4 glycosidic bonds of Glc*N*Ac, which are endogenously synthesized (Figure 7A). Lectin arrays detecting the cellular interaction of different lectins were used to characterize the changes after treating the cells with Glc*N*Al **1** and 4-*O*Me-Glc*N*Al **2** in detail. Comparing the two modified sugars, a strong reduction of 50–70% for some lectins was observed. Interestingly, the binding of some lectins was not affected.

Comparable to the literature, the lectin array shows a strong expression of oligomannose (ConA, PSA, GNA) and branched complex *N*-glycans (DSA, PHA-l, PHA-E) of these melanoma cells [45]. In addition, the polarity of the cells is confirmed due to the overexpression of terminal sialic acid-bearing glycans by binding to MAA, SNA, and ACA (Figure 7B). Thereby, for lectins such as ConA, GNA, PSA, and HHA, a strong effect was observed, as binding to the cells was significantly reduced after treatment with 4-*O*Me-Glc*N*Al **2** compared to Glc*N*Al **1**. These lectins all recognize oligomannose *N*-glycan binding motifs [62,63,64]; thus, we can conclude that high mannose epitopes are strongly reduced after treatment with 4-*O*Me-Glc*N*Al **2**, because of the inhibition of the 1-4 glycosidic linkage. Similarly, after treatment with 4-*O*Me-Glc*N*Al **2**, the expression of more complex *N*-glycan epitopes (tri- and tetra-antennary, bisecting) is drastically reduced, which is demonstrated by the binding to DSA as well as a moderate binding reduction to PHA-I and PHA-E. Evaluating the terminal Glc*N*Ac-, Chitin-, Galactose-, and *N*-Acetyllactosamine (Lac*N*Ac) binding lectins (GSL-II, WGA, BPA, and WFA), a significantly reduced cell binding was observed after treatment with 4-*O*Me-Glc*N*Al **2**. Additionally, performing stainings with fluorescence-labeled WGA showed a reduced fluorescence signal in NGI-1- and 4-*O*Me-Glc*N*Al 2-treated cells, further proving the changes in glycocalyx composition.

Regarding the terminal glucosamine-binding lectins (WGA and GSL-II), the lower fixation can be explained by the blockage of the assembly of the *N*-glycan core structure by the first β-1,4-glycosidic bond, since the lectins have a lower binding affinity for Asn-Glc*N*Ac. The evaluation of *O*-glycan binding lectins ACA, AiA, PNA, HPA, and MPA indicates a reduced expression of extended core 1, core 2, and core 3 structures. In addition, a change in polarity of the glycocalyx (sialic acid and sulfation substitution) can be assumed by the treatment of the newly generated sugar 4-*O*Me-Glc*N*Al **2**, based on the lower binding affinity to MAA and SNA-1 [61].

Cuevas et al. analyzed the clinical outcome of patients with different glycan compositions of their cell lines. Similarly to our study, the binding to different lectins was investigated and the patients were separated according to their progression-free or overall survival. In the case of patients with worse clinical outcomes, an overexpression of the TF antigen and terminal Glc*N*Ac moieties were detected. In our study, we have shown a highly reduced level in lectin binding of ACA and WGA, indicating these binding motifs. Furthermore, they showed that the overexpression of sialic acid and fucose residues results in worse clinical outcomes. Also, the strongly reduced binding to SNA and MAA indicates a decline in overexpression [45].

Additionally, it is well known that the progression of many tumors is associated with an increased expression of the α_v_β_3_ integrin, as it is involved in signal transduction and cell-to-cell interactions [65,66]. Therefore, Pochec et al. [67] investigated the alteration of the *N*-glycosylation of the integrin in melanoma cell lines during tumor progression. Thereby, both subunits of α_v_β_3_ high mannose, as well as complex glycans with bisecting Glc*N*Ac, are enriched [68,69]. While the abundance of these motifs are continuously changing during tumor progression [67,70,71,72], the treatment of 4-*O*Me-Glc*N*Al **2** indicates a highly reduced amount of oligomannose structures expressed on the cell’s membrane. Moreover, bisecting glycans seem to be less frequent, represented by the significantly reduced binding affinity to ConA and the reduced binding to GNA, PSA, HAA, and PHA-E. In addition, the promotion of melanoma migration is enhanced by β1-6-branched sialylated complex-type *N*-glycans, which is verified by the binding of primary vs. metastatic melanoma cells to SNA, and MAA [67]. We discovered a reduced binding to the two mentioned lectins by treating the cells with the *C*4-modified monosaccharide. Also, polylactosamine (polyLac*N*Ac) chains are related to increased melanoma cell motility [21]. The decreased occurrence of the polyLac*N*Ac motif is shown by the significantly reduced binding to DSA.

## 5. Conclusions

Herein, we demonstrated for the first time the successful metabolic incorporation of glucosamine derivatives additionally altered at the 4-position. We focused on a one-step approach to maximize the impact on melanoma cell behavior through the metabolic introduction of artificial, clickable derivatives that disrupt glycoprotein chain elongation. This leads to a better understanding of how the glycocalyx is involved in tumor progression and opens a new field to introduce precisely defined deficient glycan structures. Since the glycocalyx has an impact on the effects of therapeutics [73] and their efficiency, as well as cellular proliferation and metastasis, future studies need to show the therapeutic potential of the new modified sugar moieties.

## Data Availability

The lead contact will share data reported in this paper upon request.

## Supplementary Materials

The following supporting information can be downloaded at: https://www.mdpi.com/article/10.3390/cells13221831/s1.
