# Supplementary material for: Modifying the Glycocalyx of Melanoma Cells via Metabolic Glycoengineering Using N-Acetyl-d-glucosamine Analogues
Source: Cells. 2024 Nov 6;13(22):1831. doi: 10.3390/cells13221831 (PMC11592549; doi:10.3390/cells13221831)
Supplement: Supplementary file 1 [file cells-13-01831-s001.zip › cells-3266927-supplementary.pdf]

Suppl.Fig.S1

Compounds, synthetic chemistry

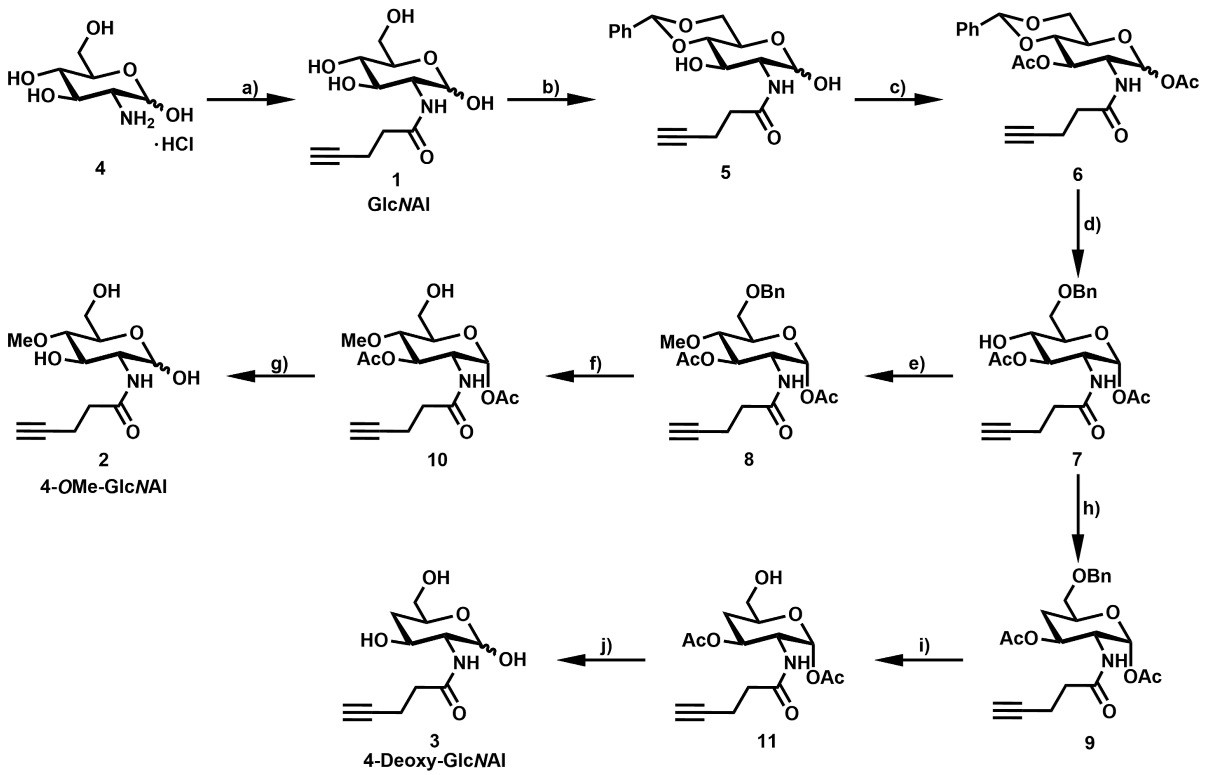

a) 2-*N*-(4'-pentynoyl)-D-glucopyranosylamine (1)
Glucosamine hydrochloride (4.00 g, 18.6 mmol) was dissolved in dry DMF (90 mL). After addition of DIPEA (4.1 mL, 24.1 mmol) and Perfluorophenyl pent-4-ynoate (5.15 g, 19.5 mmol) the mixture was stirred at ambient temperature for 18 h. Afterwards, the solvent was evaporated and the residue was washed with a DCM:MeOH mixture (5:1). The residue was further purified by column chromatography (DCM:MeOH 10:1). Glc*N*Al (mixture of anomers α/β 73:27) was isolated as a colorless solid (4.63 g, 17.9 mmol, 96%). α-anomer: **^{1}H NMR** (DMSO-d_6): δ = 7.75 (d, ^3J = 8.5 Hz, 1H, N*H*), 6.42 (d, ^3J = 4.4 Hz, O*H*), 4.95–4.87 (m, 2H, *H*-1, O*H*), 4.60 (d, ^3J = 5.6 Hz, O*H*), 4.46–4.39 (m, 1H, O*H*), 3.64–3.54 (m, 3H, *H*-2, *H*-4, *H*-6a), 3.53–3.38 (m, 2H, *H*-3, *H*-6b), 3.15–2.99 (m, 1H, *H*-5), 2.79–2.70 (m, 1H, CC*H*), 2.41–2.24 (m, 4H, CH_2) ppm. **^{13}C NMR** (DMSO-d_6): δ = 170.5 (N*C*), 90.7 (*C*-1), 84.1 (*C*CH), 72.1 (*C*-4), 71.3 (C*C*H), 71.2 (*C*-5), 70.5 (*C*-3), 61.2 (*C*-6), 54.4 (*C*-2), 34.1 (CH_2), 14.2 (CH_2) ppm.
β-anomer: **^{1}H NMR** (DMSO-d_6): δ = 7.69 (d, ^3J = 8.2 Hz, 1H, N*H*), 6.50 (d, ^3J = 6.2 Hz, 1H, O*H*), 4.95–4.87 (m, 1H, O*H*), 4.80 (d, ^3J = 5.2 Hz, 1H, O*H*), 4.52 (dd, ^3J = 6.2 Hz, ^3J = 5.5 Hz, 1H, O*H*), 4.46–4.39 (m, 1H, *H*-1), 3.71–3.64 (m, 1H, *H*-6a), 3.53–3.38 (m, 1H, *H*-6b), 3.38–2.21 (m, 2H, *H*-2, *H*-3), 3.15–2.99 (m, 2H, *H*-4, *H*-5), 2.79–2.70 (m, 1H, CC*H*), 2.41–2.24 (m, 4H, CH_2, CH_2) ppm. **^{13}C NMR** (DMSO-d_6): δ = 170.7 (N*C*), 95.5 (*C*-1), 84.0 (*C*CH), 76.9 (*C*-4), 74.4 (*C*-3), 71.4 (C*C*H), 70.8 (*C*-5), 61.2 (*C*-6), 57.3 (*C*-2), 34.6 (CH_2), 14.2 (CH_2) ppm. (Figure S1)
HRMS(ESI+) *m/z*: $[GlcNAl+Na]^+$ calcd. 282.0948, found: 282.0961 ($|\Delta m/z|$ = 4.7 ppm).

b) 2-*N*-(4'-Pentynoyl)-4,6-*O*-(phenylmethylene)-D-glucopyranosylamine (5)
Glc*N*Al (3.20 g, 12.3 mmol) was dissolved in dry MeCN (40 mL). After the addition of benzaldehyde dimethyl acetal (4.63 mL, 30.9 mmol) and camphorsulfonic acid (143 mg, 617 µmol) the reaction mixture was stirred at 35 °C for 18 h. The resulting solution was evaporated and the residue purified by column chromatography on SiO_2 with gradient elution (DCM/MeOH 20:1 → 10:1). The product was isolated as a colorless solid (3.38 g, 9.73 mmol, 79%; mixture of anomers α/β 78:22).
α-anomer: **^{1}H NMR** (DMSO-d_6): δ = 7.88 (d, ^3J = 8.3 Hz, 1H, N*H*), 7.49–7.42 (m, 2H, $H_{arom.}$), 7.41–7.35 (m, 3H, $H_{arom.}$), 6.80–6.75 (m, 1H, O*H*), 5.60 (s, 1H, O^{6}C*H*), 5.06 (d, ^3J = 5.6 Hz, 1H, O*H*), 4.97 (dd, ^3J = 4.2 Hz, ^3J = 3.8 Hz, 1H, *H*-1), 4.10 (dd, ^3J = 10.0 Hz, ^3J = 4.7 Hz, 1H, *H*-4), 3.90–3.64 (m, 4H, *H*-2, *H*-3, *H*-5, *H*-6a), 3.44 (dd, ^3J = 9.2 Hz, ^3J = 9.2 Hz, 1H, *H*-6b), 2.78–2.73 (m, 1 H, CC*H*), 2.36–2.30 (m, 4H, CH_2) ppm. **^{13}C NMR** (DMSO-d_6): δ = 170.4 (N*C*), 137.7 ($C_{arom.}$), 128.9 ($C_{arom.}$), 128.0 ($C_{arom.}$), 126.4 ($C_{arom.}$), 100.9 (O^6*C*), 91.5 (*C*-1), 83.9 (*C*CH), 82.4 (*C*-4), 71.3 (C*C*H), 68.3 (*C*-5), 67.2 (*C*-3), 62.1 (*C*-6), 54.7 (*C*-2), 34.0 (CH_2), 14.1 (CH_2) ppm. β-anomer: **^{1}H NMR** (DMSO-d_6): δ = 7.86 (d, ^3J = 9.0 Hz, 1H, N*H*), 7.49–7.42 (m, 2H, $H_{arom.}$), 7.42–7.34 (m, 3H, $H_{arom.}$), 6.80–6.75 (m, 1H, O*H*), 5.60 (s, 1H, O^{6}C*H*), 5.18 (d, ^3J = 5.5 Hz, 1H, O*H*), 4.62 (dd, ^3J = 8.3 Hz, ^3J = 6.7 Hz, 1H, *H*-1), 4.17 (dd, ^3J = 10.1 Hz, ^3J = 4.9 Hz, 1H, *H*-4), 3.90–3.64 (m, 2H, *H*-2, *H*-6a), 3.58 (ddd, ^3J = 9.5 Hz, ^3J = 9.5 Hz, ^3J = 5.6 Hz, 1H, *H*-3), 3.41 (dd, ^3J = 9.2 Hz, ^3J = 9.2 Hz, 1H, *H*-6b), 3.30 (ddd, ^3J = 9.4 Hz, ^3J = 9.4 Hz, ^3J = 4.6 Hz, 1H, *H*-5), 2.78–2.73 (m, 1 H, CC*H*), 2.36–2.30 (m, 4H, CH_2) ppm. **^{13}C NMR** (DMSO-d_6): δ = 169.2 (N*C*), 137.7 ($C_{arom.}$), 128.9 ($C_{arom.}$), 128.0 ($C_{arom.}$), 126.4 ($C_{arom.}$), 100.7 (O^6*C*), 95.6 (*C*-1), 83.6 (*C*CH), 81.5 (*C*-4), 71.3 (C*C*H), 69.8 (*C*-5), 68.8 (*C*-3),

62.2 (*C*-6), 56.5 (*C*-2), 34.5 (*C*H2), 14.2 (*C*H2) ppm. **HRMS(ESI+)** *m/z*: $[5+Na]^+$ calcd. 370.1261, found: 370.1258 ($|\Delta m/z|$ = 0.7 ppm).

c) 1,3-Di-*O*-acetyl-2-*N*-(4'-pentynoyl)-4,6-*O*-(phenylmethylene)-D-glucopyranosylamine (6)
Glucosamine derivative **5** (3.38 g, 9.73 mmol) and Ac_2O (18.4 mL, 195 mmol) were dissolved in dry pyridine (50 mL) and stirred at ambient temperature for 18 h. After addition of toluene (10 mL), the solvent was evaporated and the residue was purified by column chromatography (DCM:MeOH 30:1) to afford a colorless powder (4.19 g, 9.71 mmol, 99%; mixture of anomers α/β 77:23).
α-anomer: **^{1}H NMR** (DMSO-*d*6): δ = 8.13 (d, ^3J = 9.0 Hz, 1H, N*H*), 7.42–7.34 (m, 5H, *H*arom.), 5.93 (d, ^3J = 3.8 Hz, 1H, *H*-1), 5.69– 5.62 (m, 1H, O^6CH), 5.28–5.18 (m, 1H, *H*-3), 4.36 (ddd, ^3J = 10.7 Hz, ^3J = 9.1 Hz, ^3J = 3.7 Hz, 1H, *H*-2), 4.19 (dd, ^3J = 10.0 Hz, ^3J = 4.1 Hz, *H*-6a), 3.98–3.72 (m, 3H, *H*-5, *H*-6a, *H*-6b), 2.78–2.72 (m, 1 H, CC*H*), 2.38–2.20 (m, 4H, C*H2*), 2.19 (s, 3H, C*H3*), 2.00 (s, 3H, C*H3*) ppm. **^{13}C NMR** (DMSO-*d*6): δ = 171.1 (N*C*), 169.8 (O*C*(O)C), 169.3 (O*C*(O)C), 137.2 (*C*arom.), 129.0 (*C*arom.), 128.1 (*C*arom.), 126.1 (*C*arom.), 100.5 (O^6C), 90.3 (*C*-1), 83.4 (*C*CH), 78.1(*C*-4), 71.2 (C*C*H), 68.9 (*C*-3), 67.5 (*C*-6), 64.7 (*C*-5), 50.3 (*C*-2), 33.8 (*C*H2), 20.9 (*C*-13), 20.7 (*C*-15), 14.2 (*C*H2) ppm.
β-anomer: **^{1}H NMR** (DMSO-*d*6): δ = 8.10 (d, ^3J = 9.2 Hz, 1H, N*H*), 7.42–7.34 (m, 5H, *H*arom.), 5.75 (d, ^3J = 8.6 Hz. 1H, *H*-1), 5.69– 5.62 (m, 1H, O^6CH), 5.28–5.18 (m, 1H, *H*-3), 4.25 (dd, ^3J = 10.2 Hz, ^3J = 4.8 Hz, 1H, *H*-4), 4.04 (dd, ^2J = 19.1 Hz, ^3J = 9.2 Hz, 1H, *H*-2), 3.98–3.72 (m, 2H, *H*-6a, *H*-6b), 3.64 (ddd, ^3J = 9.7 Hz, ^3J = 9.7 Hz, ^3J = 4.8 Hz, 1H, *H*-5), 2.78–2.72 (m, 1 H, CC*H*), 2.38–2.20 (m, 4H, C*H2*), 2.03 (s, 3H, C*H3*), 1.98 (s, 3H, C*H3*) ppm.**^{13}C NMR** (DMSO-*d*6): δ = 170.5 (N*C*), 169.6 (O*C*(O)C), 168.9 (O*C*(O)C), 137.2 (*C*arom.), 129.0 (*C*arom.), 128.1 (*C*arom.), 126.1 (*C*arom.), 100.3 (O^6C), 92.3 (*C*-1), 83.3 (*C*CH), 77.7 (*C*-4), 71.2 (C*C*H), 71.2 (*C*-3), 67.3 (*C*-6), 66.4 (*C*-5), 52.4 (*C*-2), 34.3 (*C*H2), 20.6 (*C*-13), 20.5 (*C*-15), 14.2 (*C*H2) ppm. (Figure S2) **HRMS(ESI+)** *m/z*: $[6+Na]^+$ calcd. 454.1472 , found: 454.1476 ($|\Delta m/z|$ = 0.8 ppm).

d) 1,3-Di-*O*-acetyl-2-*N*-(4'-pentynoyl)-6-*O*-(phenylmethyl)-α-D-glucopyranosylamine (7)
At 0 °C the acetal **6** (3.00 g, 6.95 mmol) was dissolved in dry DCM (12 mL). After the addition of Trifluoroacetic anhydride (967 µL, 6.95 mmol) and triethylsilane (6.66 mL, 41.7 mmol) the solution was stirred for 10 min. Dropwise TFA (2.68 mL, 34.8 mmol) was added over 1 h and the mixture was stirred for further 3.5 h. The reaction mixture was neutralized using NEt_3 (12 mL) and washed with a saturated $NaHCO_3$ solution. The organic layer was dried over $MgSO_4$ and the solvent was removed. The residue was purified by column chromatography (DCM:MeOH 50:1) to afford the product as a colorless foam (2.38 g, 5.49 mmol, 79%)
^{1}H NMR ($CDCl_3$): δ = 7.39–7.28 (m, 5H, *H*arom.), 6.14 (d, ^3J = 3.8 Hz, 1H, *H*-1), 5.81 (d, ^3J = 9.3 Hz, 1H, N*H*), 5.15 (dd, ^3J = 11.0 Hz, ^3J = 9.1 Hz, 1H, *H*-3), 4.59 (t, ^3J = 14.4 Hz, 1H, O^6CHa), 4.56 (t, ^3J = 14.4 Hz, 1H, O^6CHb), 4.38 (ddd, ^3J = 11.1 Hz, ^3J = 8.9 Hz, ^3J = 3.6 Hz, 1H, *H*-2), 3.94–3.78 (m, 3H, *H*-4, *H*-5, *H*-6a), 3.70–3.63 (m, 1H, *H*-6b), 2.96 (br, 1H, O*H*), 2.49–2.45 (m, 2H, C*H2*), 2.37– 2.32 (m, 2H, C*H2*), 2.16 (s, 3H, C*H3*), 2.12 (s, 3H, C*H3*), 1.97 (t, ^4J = 2.6 Hz, 1H, CC*H*) ppm. **^{13}C NMR** ($CDCl_3$): δ = 172.2 (O*C*(O)C), 171.0 (N*C*), 169.0 (O*C*(O)C), 137.2 (*C*arom.), 128.5 (*C*arom.), 128.0 (*C*arom.), 127.8 (*C*arom.), 90.8 (*C*-1), 82.5 (*C*CH), 73.9 (O^6C), 72.7 (*C*-3), 71.5 (*C*-5), 70.2 (*C*-4), 70.1 (*C*-6), 69.5 (C*C*H), 50.9 (*C*-2), 35.1 (*C*H2), 21.0 (*C*-13), 21.0 (*C*-15), 14.7 (*C*H2) ppm. (Figure S3) **HRMS(ESI+)** *m/z*: $[7+Na]^+$ calcd. 456.1629 , found: 456.1609 ($|\Delta m/z|$ = 4.5 ppm).

e) 4-Methoxy-1,3-Di-*O*-acetyl-2-*N*-(4'-pentynoyl)-6-*O*-(phenylmethyl)-α-D-glucopyranosylamine (8)
Compound **7** (1.97 g, 4.54 mmol) was diluted in dry DCM (40 mL) and cooled to 0 °C. After the addition of HBF_4 (wt. 50% in H_2O, 656 µL, 5.23 mmol) and 1.8 M (trimethylsilyl)diazomethane solution in hexanes (2.52 mL, 4.54 mmol), another three times every 15 min 1.8 M (trimethylsilyl)diazomethane solution in hexanes (1.26 mL, 2.27 mmol) was added. The reaction mixture was allowed to warm up to ambient temperature and stirred for 18 h. An incomplete consummation of the starting material was obtained on TLC (whether a longer reaction time, heating up nor the addition of reactants Improved the yield). After neutralization with NEt_3 (14 mL) the solution was washed with water (40 mL) and dried over $MgSO_4$. The solvent was removed under reduced pressure and the residue was purified by column chromatography (Cy:EA 5:1 → 2:1). Thereby the product was obtained as a colorless foam. Afterwards the column was flushed with pure ethyl acetate (EA) to obtain educt residues. The same procedure was performed a second time with the educt fraction and the isolated product fractions were combined (874 mg, 1.95 mmol, 43%).
^{1}H NMR ($CDCl_3$): δ = 7.38–7.27 (m, 5H, *H*arom.), 6.16 (d, ^3J = 3.6 Hz, 1H, *H*-1), 5.80 (d, ^3J = 9.0 Hz, 1H, N*H*), 5.18 (dd, ^3J = 11.1 Hz, ^3J = 9.2 Hz, 1H, *H*-3), 4.65 (d, ^2J = 12.1 Hz, 1H, O^6CHa), 4.52 (d, ^2J = 12.2 Hz, 1H, O^6CHb), 4.38 (ddd, ^3J = 11.0 Hz, ^3J = 8.9 Hz, ^3J = 3.6 Hz, 1H, *H*-2), 3.77–3.70 (m, 2H, *H*-5, *H*-6a), 3.68–3.58 (m, 2H, *H*-4, *H*-6b), 3.42 (s, 3H, OC*H3*), 2.50–2.43 (m, 2H, C*H2*), 2.36– 2.30 (m, 2H, C*H2*), 2.14 (s, 3H, C*H3*), 2.11 (s, 3H, C*H3*), 1.97 (t, ^4J = 2.7 Hz, 1H, CC*H*) ppm. **^{13}C NMR** ($CDCl_3$): δ = 171.8 (O*C*(O)C), 170.9 (N*C*), 168.9 (O*C*(O)C), 137.8 (*C*arom.), 128.4 (*C*arom.), 127.8 (*C*arom.), 127.7 (*C*arom.), 91.0 (*C*-1), 82.5 (*C*CH), 76.5 (*C*-4), 73.6 (O^6C), 73.2 (*C*-3), 72.8 (*C*-5), 69.4 (C*C*H), 67.8 (*C*-6), 60.7 (O*C*H3), 51.3 (*C*-2), 35.1 (*C*H2), 21.1 (*C*-15), 21.0 (*C*-13), 14.6 (*C*H2) ppm. (Figure S4)
HRMS(ESI+) *m/z*: $[5+Na]^+$ calcd. 470.1785 , found: 470.1771 ($|\Delta m/z|$ = 3.1 ppm).

f) 4-Methoxy-1,3-Di-*O*-acetyl-2-*N*-(4'-pentynoyl)-α-D-glucopyranosylamine (10)
Compound **8** (587 mg, 1.31 mmol) and DDQ (1.49 g, 6.56 mmol) were dissolved in dry DCM (40 mL) and stirred for 2 d at 44 °C. After addition of water (10 mL) and additional stirring for 30 min the organic layer was separated and washed with saturated $NaHCO_3$ solution and Brine. The solvent was removed under reduced pressure and the crude product was purified by column chromatography (DCM:MeOH 100:1 → 50:1) to give a colorless oil (291 mg, 814 µmol, 62%).
^{1}H NMR ($CDCl_3$): δ = 6.12 (d, ^3J = 3.6 Hz,1H, *H*-1), 5.93 (d, ^3J = 8.9 Hz, 1H, N*H*), 5.20 (dd, ^3J = 11.2 Hz,^3J = 9.3 Hz, 1H, *H*-3), 4.34 (ddd, ^3J = 11.1 Hz,^3J = 8.8 Hz, ^3J = 3.7 Hz, 1H, *H*-2), 3.83 (dd, ^2J = 12.4 Hz,^3J = 2.5 Hz, 1H, *H*-6a), 3.74 (dd, ^2J = 12.4 Hz,^3J = 3.3 Hz, 1H, *H*-6b), 3.69–3.63 (m, 1H, *H*-5), 3.55 (d, ^3J = 9.3 Hz, 1H, *H*-4), 3.51 (s, 3H, OC*H3*), 2.50–2.43 (m, 2H, C*H2*), 2.37–2.31 (m, 2H, C*H2*), 2.14 (s, 3H, C*H3*), 2.12 (s, 3H, C*H3*), 1.97 (t, ^4J = 2.6 Hz, 1H, CC*H*) ppm. **^{13}C NMR** ($CDCl_3$): δ = 171.8 (O*C*(O)C), 171.1 (N*C*), 169.1 (O*C*(O)C), 90.8 (*C*-1), 82.5 (*C*CH), 76.4 (*C*-4), 73.2 (*C*-5), 73.0 (*C*-3), 69.4 (C*C*H), 61.0 (*C*-6), 60.8 (O*C*H3), 51.4 (*C*-2), 35.1 (*C*H2), 21.1 (*C*-15), 20.9 (*C*-13), 14.6 (*C*H2) ppm. (Figure S5)
HRMS(ESI+) *m/z*: $[10+Na]^+$ calcd. 380.1316, found: 380.1303 ($|\Delta m/z|$ = 3.4 ppm).

g) 4-Methoxy-2-*N*-(4'-Pentynoyl)-D-glucopyranosylamine (2)
To a solution of **10** (137 mg, 383 µmol) in dry MeOH (15 mL), a 0.5 M NaOMe solution (766 µL, 383 µmol) was added, and the mixture was stirred at ambient temperature for 1 h. The reaction was stopped by adding acidic ion exchange resin Amberlite until the pH was 7. The resin was filtered off and the solvent was removed *in vacuo*. After purification of the crude product by flash chromatography (RP18, H_2O with 0.1% TFA) and subsequent lyophilization a colorless solid (52.0 mg, 190 µmol, 50%; mixture of anomers α/β 70:30) was obtained.
α-anomer: **^{1}H NMR** (DMSO-d_6): δ = 7.75 (d, ^{3}J = 7.8 Hz, 1H, N*H*), 6.47 (d, ^{3}J = 4.7 Hz, 1H, O*H*), 4.90 (dd, ^{3}J = 4.5 Hz, ^{3}J = 2.5 Hz, 1H, *H*-1), 4.81 (d, ^{3}J = 6.1 Hz, 1H, O*H*), 4.58 (dd, ^{3}J = 6.6 Hz, ^{3}J = 4.9 Hz, 1H, O*H*), 3.67–3.43 (m, 5H, *H*-2, *H*-3, *H*-5, *H*-6a, *H*-6b), 3.43 (s, 3H, OC*H*3), 3.01–2.94 (m, 1H, *H*-4), 2.76–2.73 (m, 1H, CC*H*), 2.38–2.26 (m, 4H, C*H2*) ppm. **^{13}C NMR** (DMSO-d_6): δ = 170.5 (N*C*), 90.5 (*C*-1), 84.0 (*C*CH), 80.2 (*C*-4), 71.3 (C*C*H), 70.9 (*C*-5), 70.3 (*C*-3), 60.6 (*C*-6), 59.8 (O*C*H3), 54.6 (*C*-2), 34.0 (*C*H2), 14.1 (*C*H2) ppm. *β*-anomer: **^{1}H NMR** (DMSO-d_6): δ = 7.82 (d, ^{3}J = 8.2 Hz, 1H, N*H*), 6.56 (d, ^{3}J = 6.1 Hz, 1H, O*H*), 4.98 (d, ^{3}J = 5.8 Hz, 1H, O*H*), 4.67 (dd, ^{3}J = 6.4 Hz, ^{3}J = 5.1 Hz, 1H, O*H*), 4.42 (dd, ^{3}J = 7.7 Hz, ^{3}J = 6.4 Hz, 1H, *H*-1), 3.65–3.37 (m, 5H, *H*-2, *H*-3, *H*-5, *H*-6a, *H*-6b), 3.42 (s, 3H, OC*H*3), 3.09–3.03 (m, 1H, *H*-4), 2.77–2.75 (m, 1H, *CCH*), 2.38–2.26 (m, 4H, C*H2*) ppm. **^{13}C NMR** (DMSO-d_6): δ = 170.6 (N*C*), 95.3 (*C*-1), 83.9 (*C*CH), 79.9 (*C*-4), 75.5 (*C*-5), 74.1 (*C*-3), 71.3 (C*C*H), 60.6 (*C*-6), 59.7 (O*C*H3), 57.5 (*C*-2), 34.5 (*C*H2), 14.1 (*C*H2) ppm.(Figure S6)
HRMS(ESI+) *m/z*: [4-*O*Me-Glc*N*Al+Na]$^{+}$ calcd. 296.1105 , found: 296.1102 (|Δ*m/z*| = 0.9 ppm).

h) 4-Deoxy-1,3-Di-*O*-acetyl-2-*N*-(4'-pentynoyl)-6-*O*-(phenylmethyl)-α-D-glucopyranosylamine (9)
In dry toluene (80 mL) 7 (2.10 g, 2.84 mmol) was dissolved together with 1,1-(thiocarbonyl)diimidazole (1.03 g, 5.81 mmol) and refluxed at 110 °C for 2 h. After the disappearance of the starting material on TLC, Bu_3SnH (2.61 mL, 9.69 mmol) and AIBN (79.6 mg, 484 µmol) were added, and the mixture was stirred for additional 30 min at 110 °C. The reaction mixture was cooled and diluted with EA. After filtration over silica gel, the mixture was washed with saturated KF solution three times. The organic layer was dried with $MgSO_4$ and the solvent was removed. The crude product was purified by column chromatography (Cy:EA 5:1 → 3:1) to give the deoxy compound 6 as a yellow oil (1.10 g, 2.63 mmol, 54%).
^{1}H NMR ($CDCl_3$): δ = 7.37–7.27 (m, 5H, *H*arom.), 6.21 (d, ^{3}J = 3.6 Hz, 1H, *H*-1), 5.71 (d, ^{3}J = 8.9 Hz, 1H, N*H*), 5.20 (ddd, ^{3}J = 11.2 Hz, ^{3}J = 11.2 Hz, ^{3}J = 4.8 Hz, 1H, *H*-3), 4.59–4.50 (m, 2H, O^{6}C*H2*), 4.33 (ddd, ^{3}J = 10.8 Hz, ^{3}J = 9.0 Hz, ^{3}J = 3.6 Hz, 1H, *H*-2), 4.06 (dddd, ^{3}J=12.0Hz,^{3}J=4.9Hz, ^{3}J=4.9Hz,^{3}J=2.3Hz, 1H, *H*-5), 3.56 (dd, ^{2}J=10.3Hz, ^{3}J=4.8Hz, 1H, *H*-6a), 3.49 (dd, ^{2}J=10.3Hz, ^{3}J = 4.9 Hz, 1H, *H*-6b), 2.51–2.45 (m, 2H, C*H2*), 2.38–2.32 (m, 2H, C*H2*), 2.19–2.11 (m, 4H, *H*-4equ., C*H3*), 2.06 (s, 3H, C*H3*), 1.98 (t, ^{4}J = 2.6 Hz, 1H, CC*H*), 1.81 (ddd, ^{2}J = 12.6 Hz, ^{3}J = 12.1, ^{3}J = 12.0 Hz, 1H, *H*-4ax.) ppm. **^{13}C NMR** ($CDCl_3$): δ = 171.6 (O*C*(O)C), 171.0 (N*C*), 169.1 (O*C*(O)C), 137.8 (*C*arom.), 128.4 (*C*arom.), 127.8 (*C*arom.), 127.7 (*C*arom.), 91.9 (*C*-1), 82.6 (*C*CH), 73.6 (O^{6}*C*), 71.7 (*C*-6), 69.5 (C*C*H), 69.2 (*C*-5), 67.9 (*C*-3), 51.8 (*C*-2), 35.2 (*C*H2), 33.1 (*C*-4), 21.1 (*C*-15), 21.1 (*C*-13), 14.7 (*C*H2) ppm. (Figure S7) **HRMS(ESI+)** *m/z*: [9+Na]$^{+}$ calcd. 440.1680, found: 440.1679 (|Δ*m/z*| = 0.1 ppm).

i) 4-Deoxy-1,3-Di-*O*-acetyl-2-*N*-(4'-pentynoyl)-α-D-glucopyranosylamine (11)
Compound **9** (743 mg, 1.78 mmol) and DDQ (2.02 g, 8.90 mmol) were dissolved in dry DCM (30 mL) and stirred for 2 d at 44 °C. After addition of water (30 mL) and additional stirring for 30 min the organic layer was separated and washed with saturated $NaHCO_3$ solution and Brine. The solvent was removed under reduced pressure and the crude product was purified by column chromatography (DCM:MeOH 100:1 → 50:1) to give a colorless oil (362 mg, 1.11 mmol, 62%).
^{1}H NMR ($CDCl_3$): δ = 6.19 (d, ^{3}J = 3.6 Hz, 1H, *H*-1), 5.75 (d, ^{3}J = 8.9 Hz, 1H, N*H*), 5.22 (ddd, ^{3}J = 11.2 Hz, ^{3}J = 11.2 Hz, ^{3}J = 5.0 Hz, 1H, *H*-3), 4.31 (ddd, ^{3}J = 10.8 Hz,^{3}J = 9.0 Hz,^{3}J = 3.6 Hz, 1H, *H*-2), 3.98 (dddd, ^{3}J = 12.0 Hz,^{3}J = 5.7 Hz, ^{3}J = 2.9 Hz,^{3}J = 2.9 Hz, 1H, *H*-5), 3.68 (dd, ^{2}J = 11.9 Hz, ^{3}J = 3.0 Hz, 1H, *H*-6a), 3.49 (dd, ^{2}J = 12.0 Hz, ^{3}J = 5.7 Hz, 1H, *H*-6b), 2.51–2.45 (m, 2H, C*H2*), 2.38–2.32 (m, 2H, C*H2*), 2.15 (s, 3H, C*H3*), 2.06 (s, 3H, C*H3*), 2.01 (ddd, ^{2}J = 13.3 Hz, ^{3}J = 5.4, ^{3}J = 2.8 Hz, 1H, *H*-4equ.), 1.98 (t, ^{4}J = 2.6 Hz, 1H, CC*H*), 1.81 (m, 1H, *H*-4ax.) ppm. **^{13}C NMR** ($CDCl_3$): δ = 171.6 (O*C*(O)C), 171.1 (N*C*), 169.2 (O*C*(O)C), 91.8 (*C*-1), 82.6 (*C*CH), 70.5 (*C*-5), 69.5 (C*C*H), 67.7 (*C*-3), 64.6 (*C*-6), 51.7 (*C*-2), 35.2 (*C*H2), 31.9 (*C*-4), 21.1 (*C*-15), 21.0 (*C*-13), 14.7 (*C*H2) ppm. (Figure S8) **HRMS(ESI+)** *m/z*: [11+Na]$^{+}$ calcd. 350.1210, found: 350.1194 (|Δ*m/z*| = 4.6 ppm).

j) 4-Deoxy-2-*N*-(4'-pentynoyl)-D-glucopyranosylamine (3)
To a solution of 11 (166 mg, 507 µmol) in dry MeOH (10 mL), a 0.5 M NaOMe solution (1.01 mL, 507 µmol) was added and the mixture was stirred at ambient temperature for 1 h. The reaction was stopped by adding acidic ion exchange resin Amberlite until the pH was 7. The resin was filtered off and the solvent was removed *in vacuo*. After purification of the crude product by flash chromatography (RP18, H_2O with 0.1% TFA → H_2O:MeCN = 9:1 with 0.1% TFA) and subsequent lyophilization a colorless solid (89.2 mg, 367 µmol, 72%; mixture of anomers α/β 78:22) was obtained.
α-anomer: **^{1}H NMR** (CD_3OD): δ = 5.14 (d, ^{3}J = 3.4 Hz, 1H, *H*-1), 4.07 (dddd, ^{3}J = 11.9 Hz,^{3}J = 5.0 Hz,^{3}J = 5.0 Hz,^{3}J = 2.1 Hz,1H, *H*- 5), 3.92 (ddd, ^{3}J = 10.7 Hz, ^{3}J = 10.7 Hz, ^{3}J = 4.6 Hz, 1H, *H*-3), 3.79 (dd, ^{3}J = 10.4 Hz, ^{3}J = 3.5 Hz, 1H, *H*-2), 3.55–3.52 (m, 2H, *H*-6a, *H*- 6b), 2.50–2.45 (m, 4H, C*H2*), 2.28–2.24 (m, 1H, CC*H*), 1.99 (ddd, ^{3}J = 12.6 Hz, ^{3}J = 4.9 Hz, ^{3}J = 2.3 Hz, 1H, *H*-4equ.), 1.42 ('vq', *J* = 12.0 Hz, 1H, *H*-4ax.) ppm. **^{13}C NMR** (CD_3OD): δ = 174.5 (N*C*), 93.2 (*C*-1), 83.6 (*C*CH), 70.2 (C*C*H), 69.4 (*C*-5), 66.8 (*C*-3), 65.9 (*C*-6), 57.5 (*C*-2), 37.5 (*C*-4), 36.0 (*C*H2), 15.6 (*C*H2) ppm.
β-anomer: **^{1}H NMR** (CD_3OD): δ = 4.51 (d, ^{3}J = 8.3 Hz, 1H, *H*-1), 3.71–3.60 (m, 1H, *H*-3), 3.59–3.50 (m, 3H, *H*-5, *H*-6), 3.45 (dd, ^{3}J = 10.0 Hz, ^{3}J = 8.2 Hz, 1H, *H*-2), 2.50–2.45 (m, 4H, C*H2*), 2.28–2.24 (m, 1H, CC*H*), 2.03–1.94 (m, 1H, *H*-4equ.), 1.48–1.28 (m, 1H, *H*- 4ax.) ppm. **^{13}C NMR** (CD_3OD): δ = 174.5 (N*C*), 97.4 (*C*-1), 83.7 (*C*CH), 74.0 (*C*-5), 70.6 (*C*-3), 70.1 (C*C*H), 65.6 (*C*-6), 60.5 (*C*-2), 37.1 (*C*-4), 36.4 (*C*H2), 15.6 (*C*H2) ppm. (Figure S9) **HRMS(ESI+)** *m/z*: [4-Deoxy-Glc*N*Al+Na]$^{+}$ calcd. 266.0999, found: 266.0997 (|Δ*m/z*| = 0.8 ppm).

Suppl. Figures

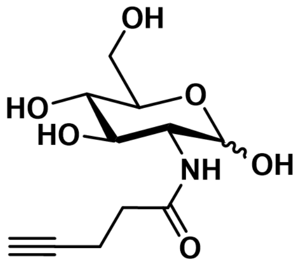

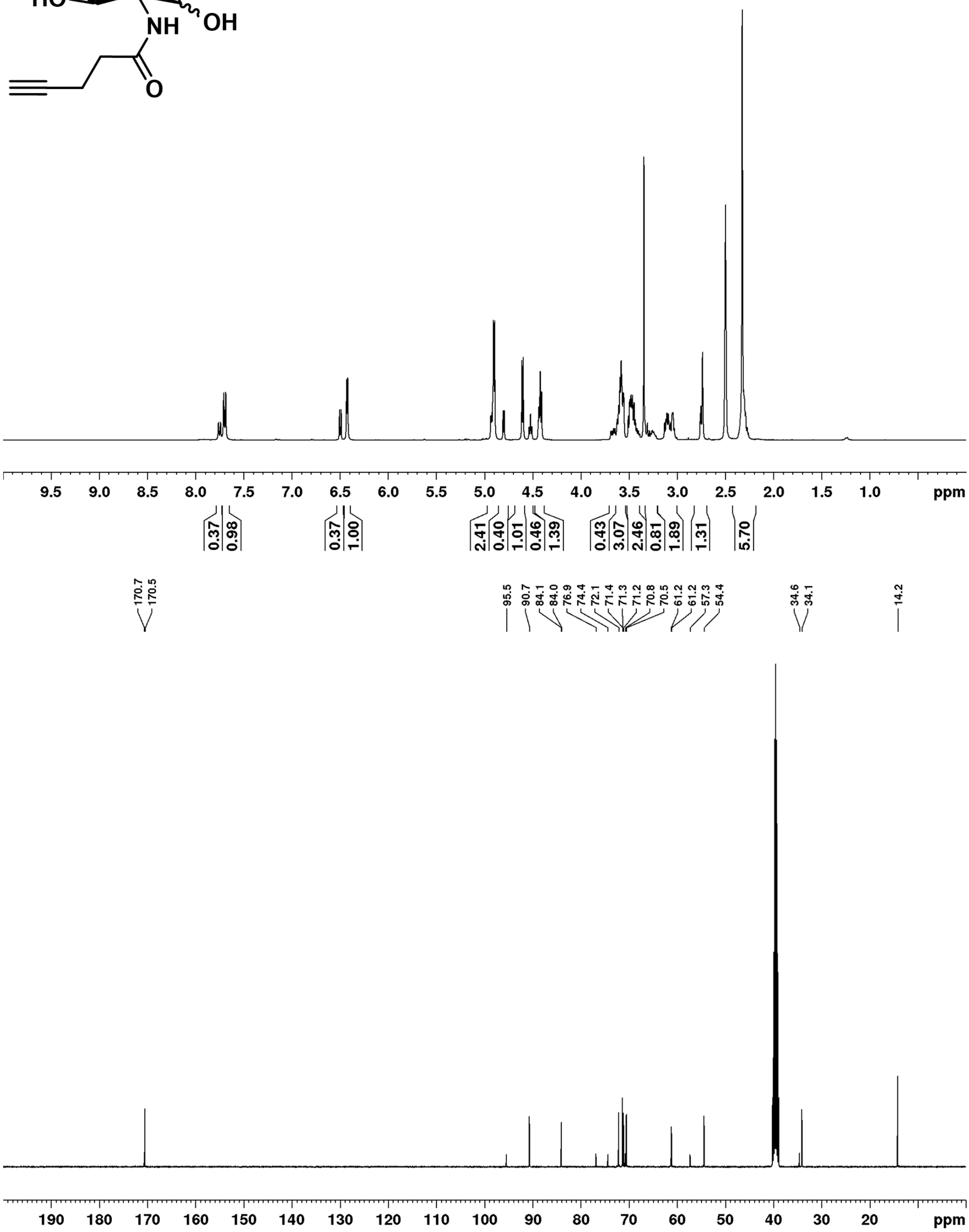

^{1}H NMR (400 MHz, $CDCl_3$) and ^{13}C NMR (100 MHz, $CDCl_3$) spectra of compound **Glc*N*Al**.

Suppl. Figures

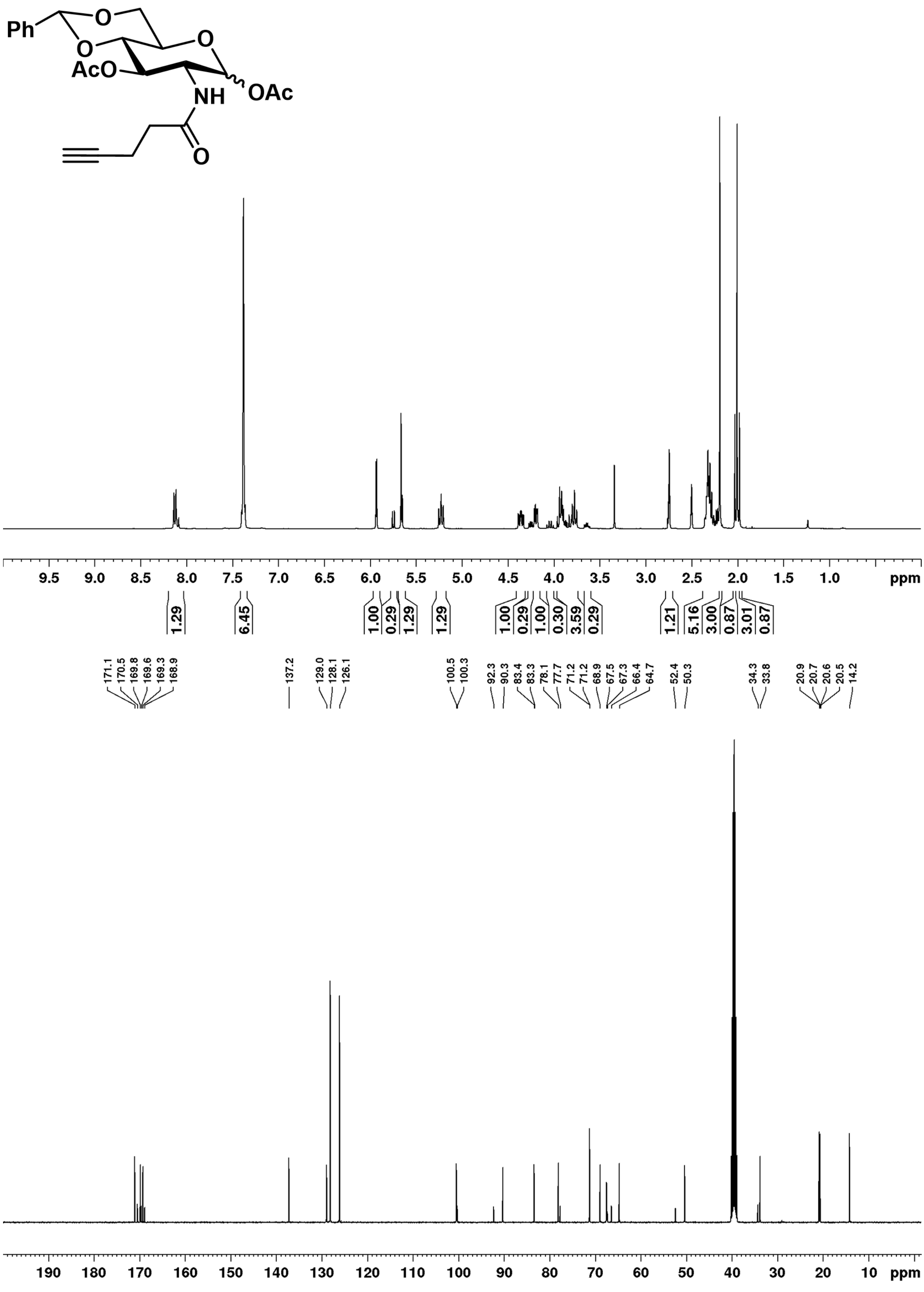

^{1}H NMR (400 MHz, $CDCl_3$) and ^{13}C NMR (100 MHz, $CDCl_3$) spectra of compound **6**.

Suppl. Figures

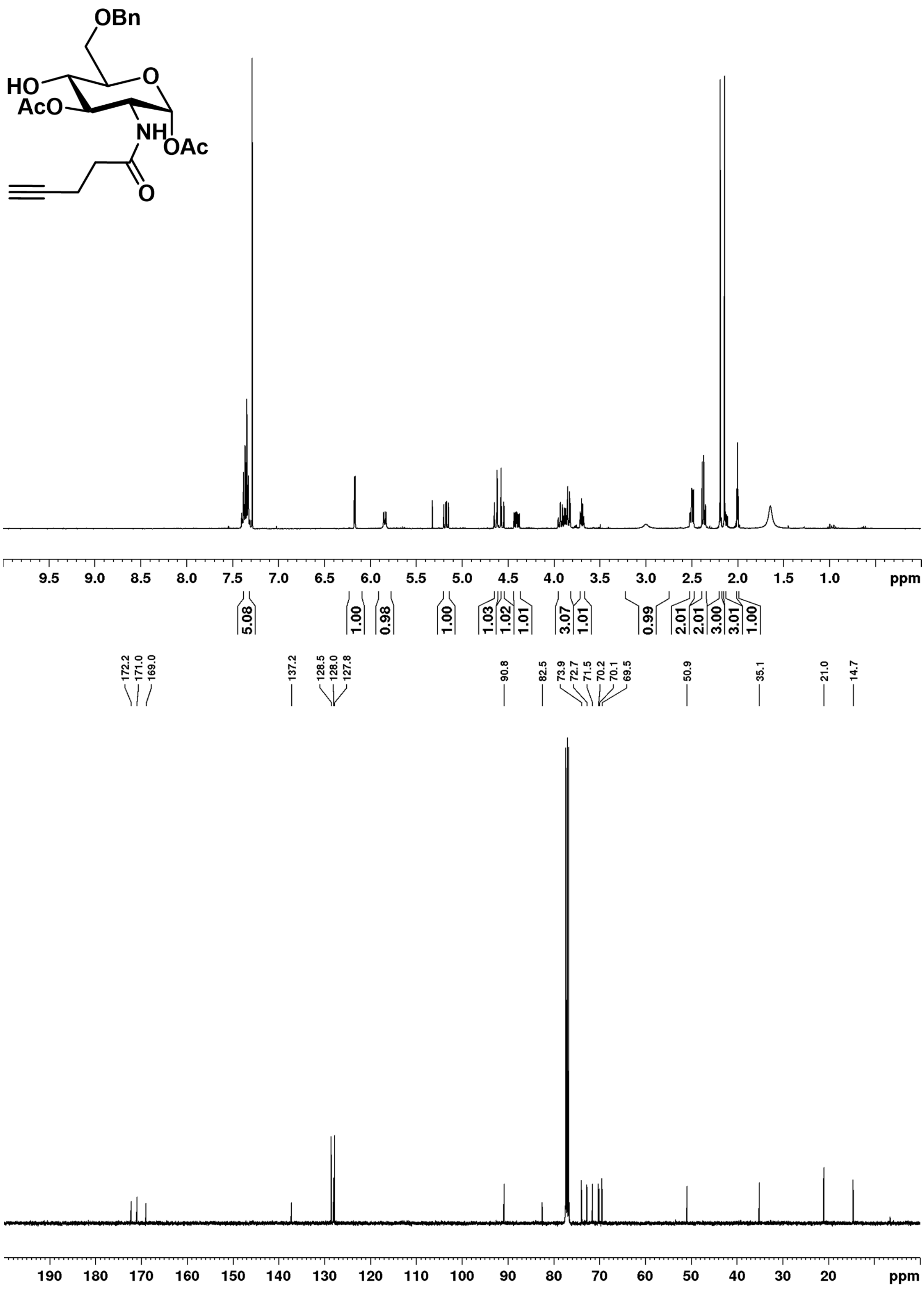

^{1}H NMR (400 MHz, $CDCl_3$) and ^{13}C NMR (100 MHz, $CDCl_3$) spectra of compound **7**.

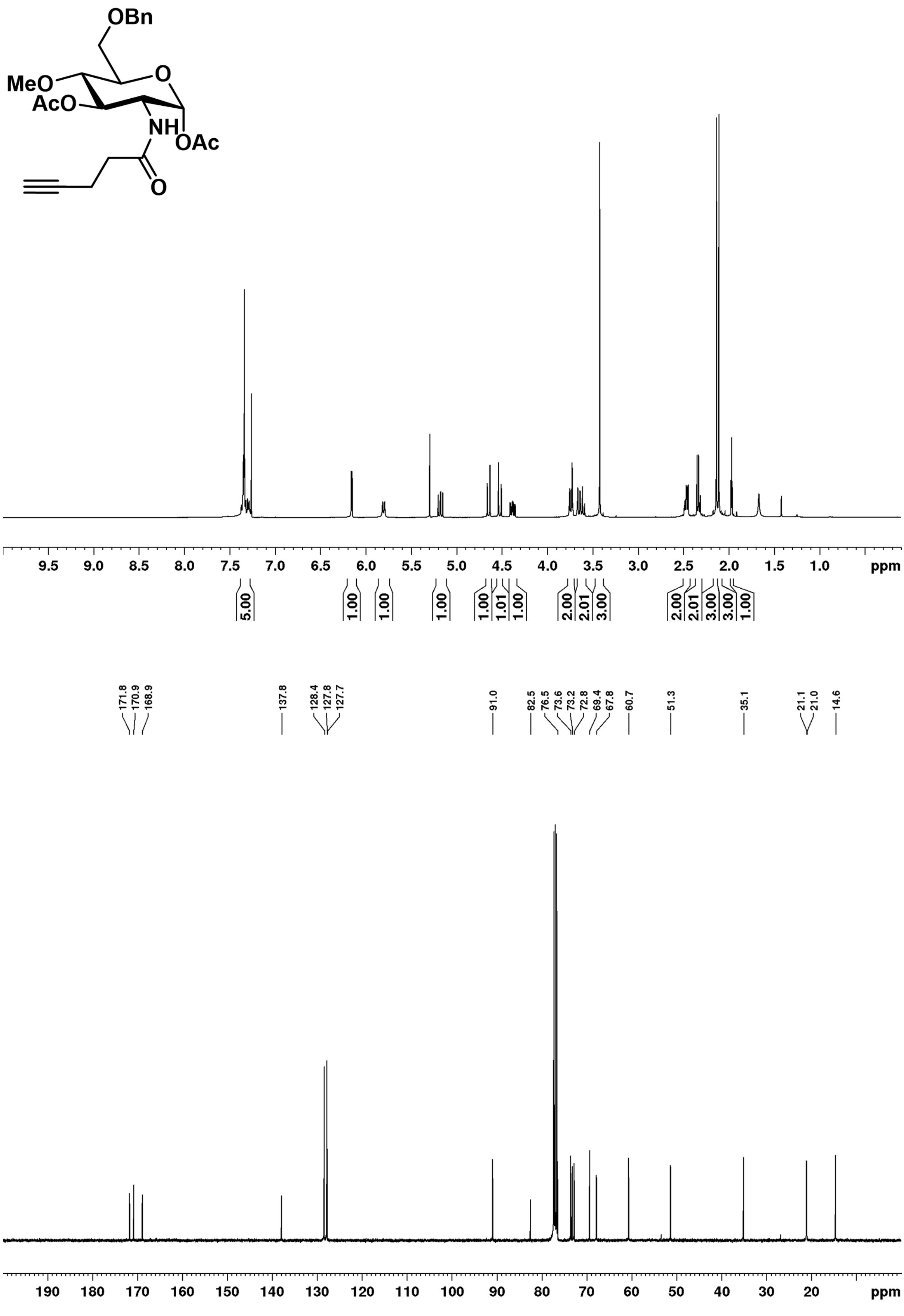

^{1}H NMR (400 MHz, $CDCl_3$) and ^{13}C NMR (100 MHz, $CDCl_3$) spectra of compound **8**.

Suppl. Figures

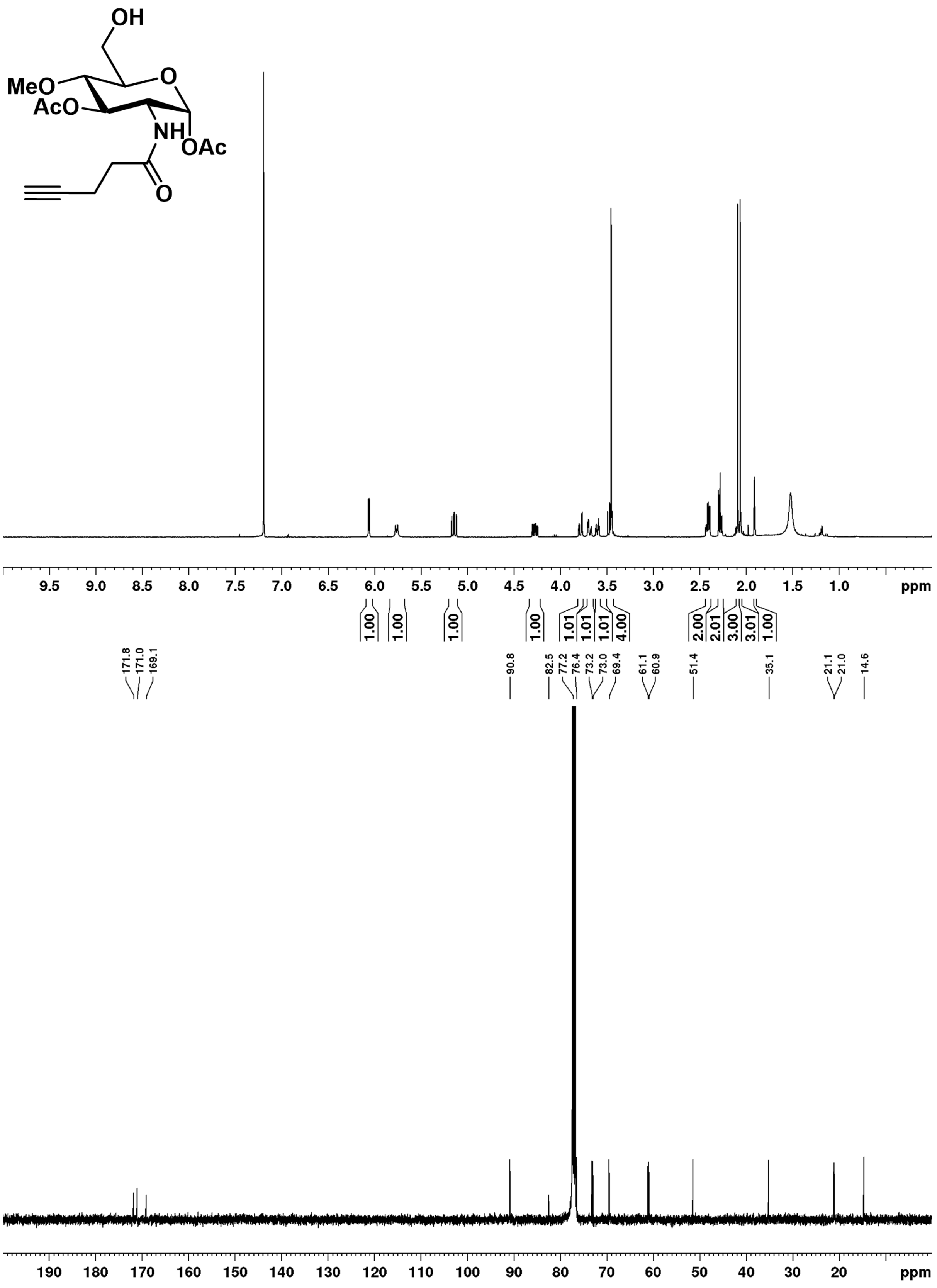

^{1}H NMR (400 MHz, $CDCl_3$) and ^{13}C NMR (100 MHz, $CDCl_3$) spectra of compound **10**.

Suppl. Figures

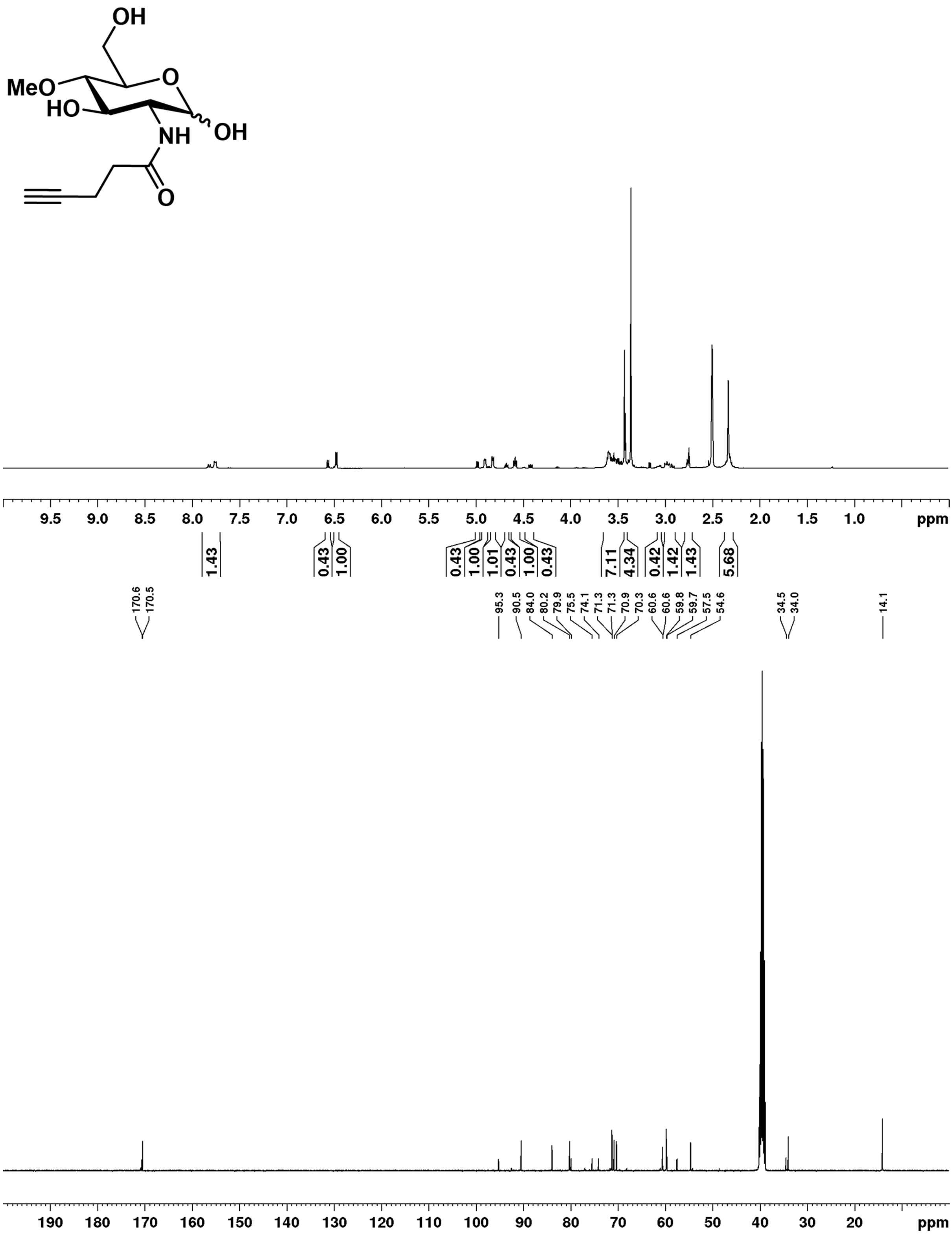

^{1}H NMR (400 MHz, DMSO-d_6) and ^{13}C NMR (100 MHz, DMSO-d_6) spectra of compound **2**.

Suppl. Figures

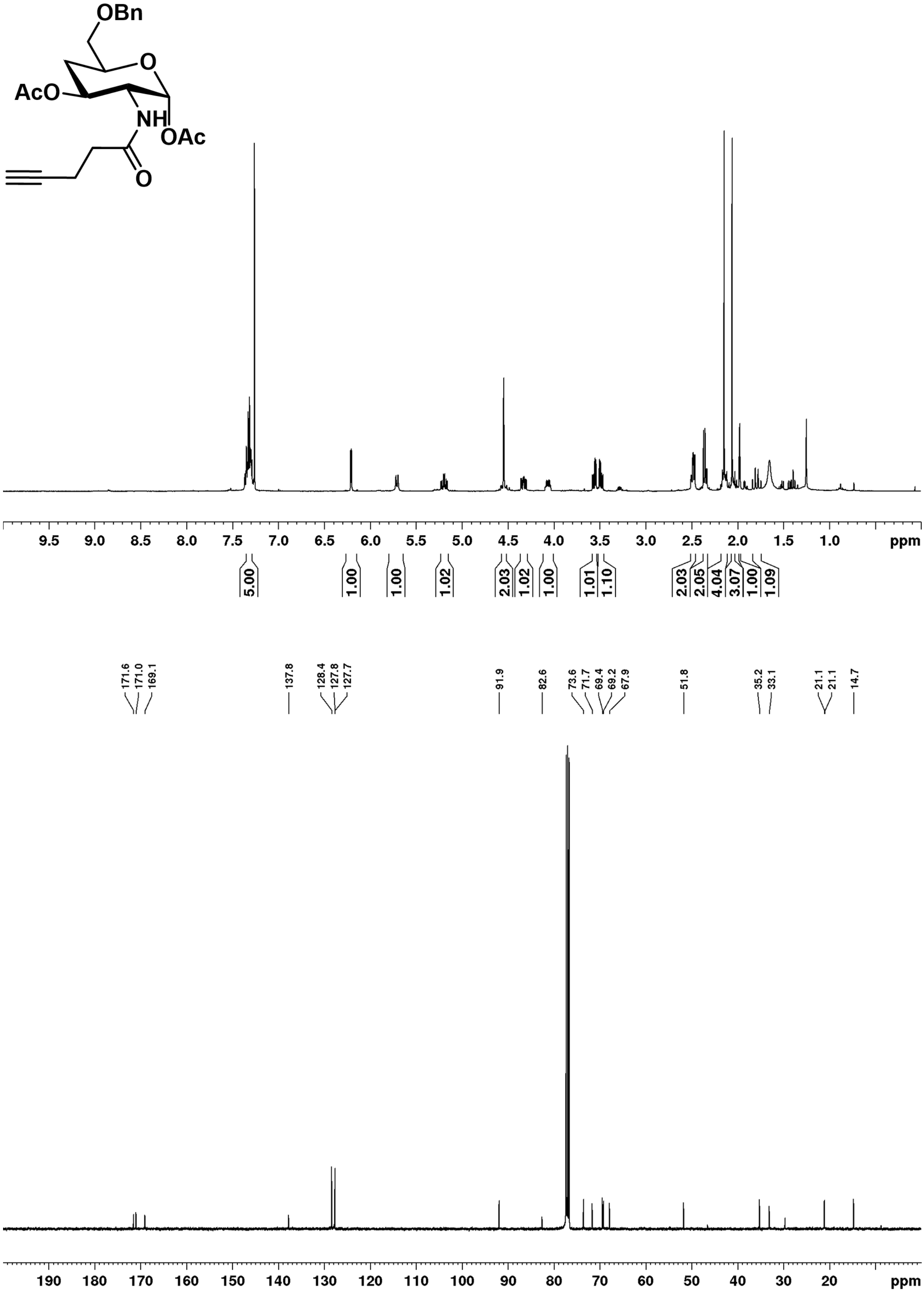

^{1}H NMR (400 MHz, $CDCl_3$) and ^{13}C NMR (100 MHz, $CDCl_3$) spectra of compound **9**.

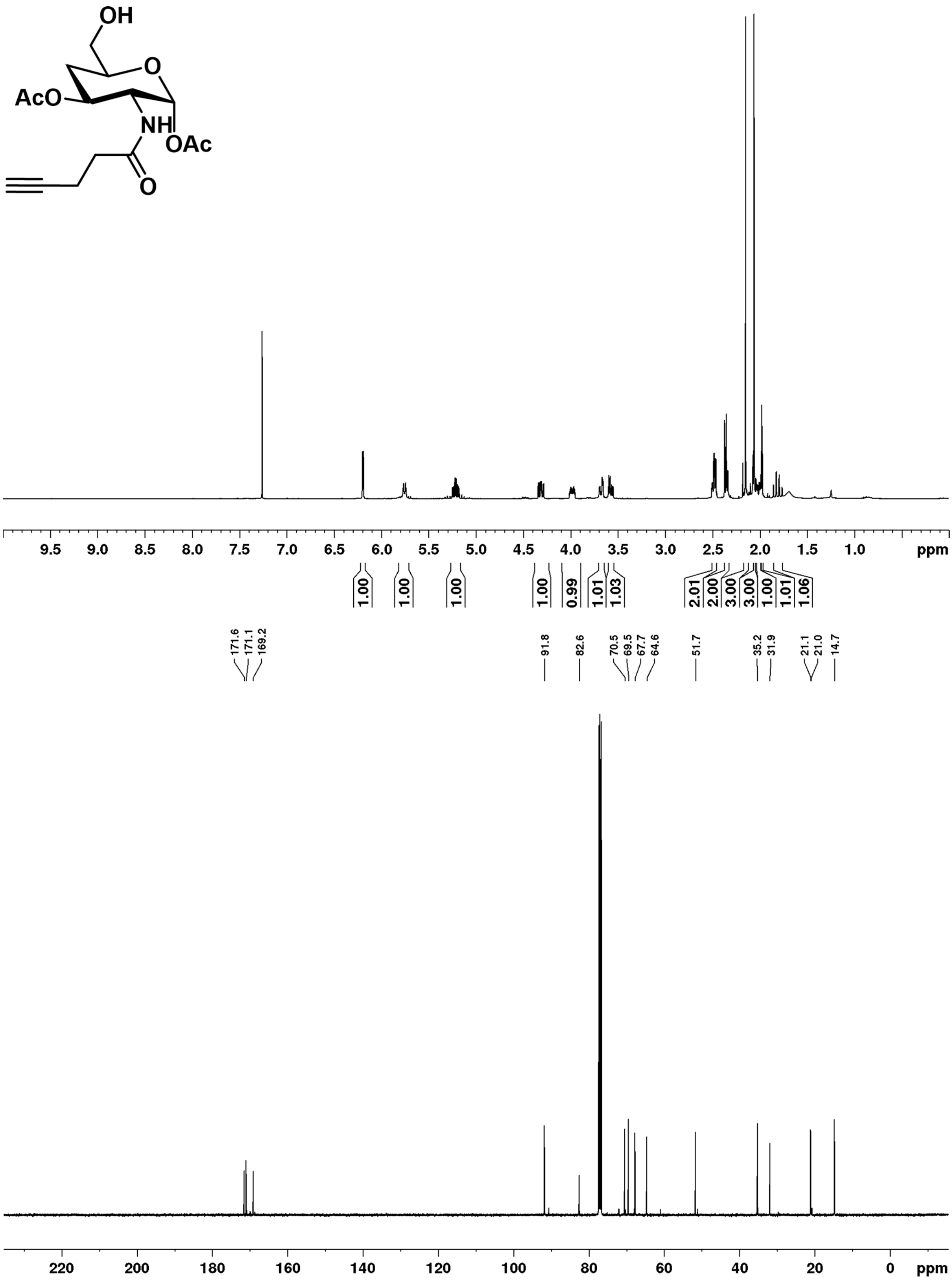

^{1}H NMR (400 MHz, $CDCl_3$) and ^{13}C NMR (100 MHz, $CDCl_3$) spectra of compound **11**.

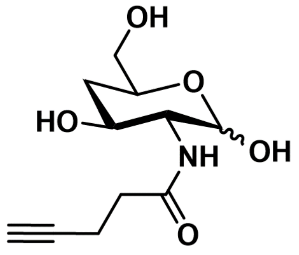

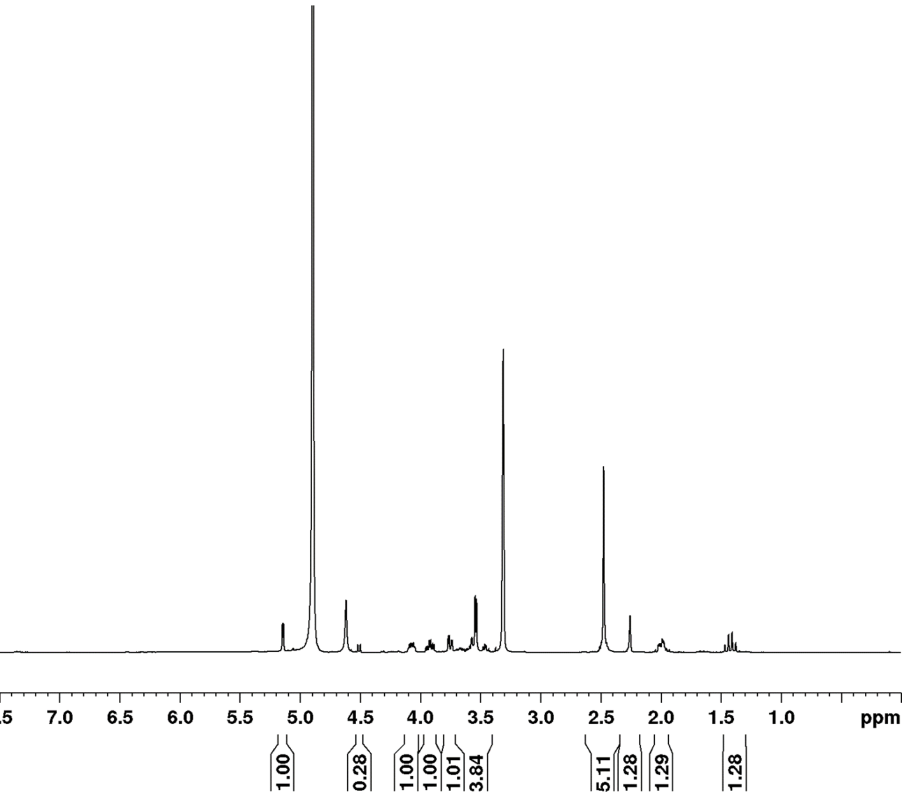

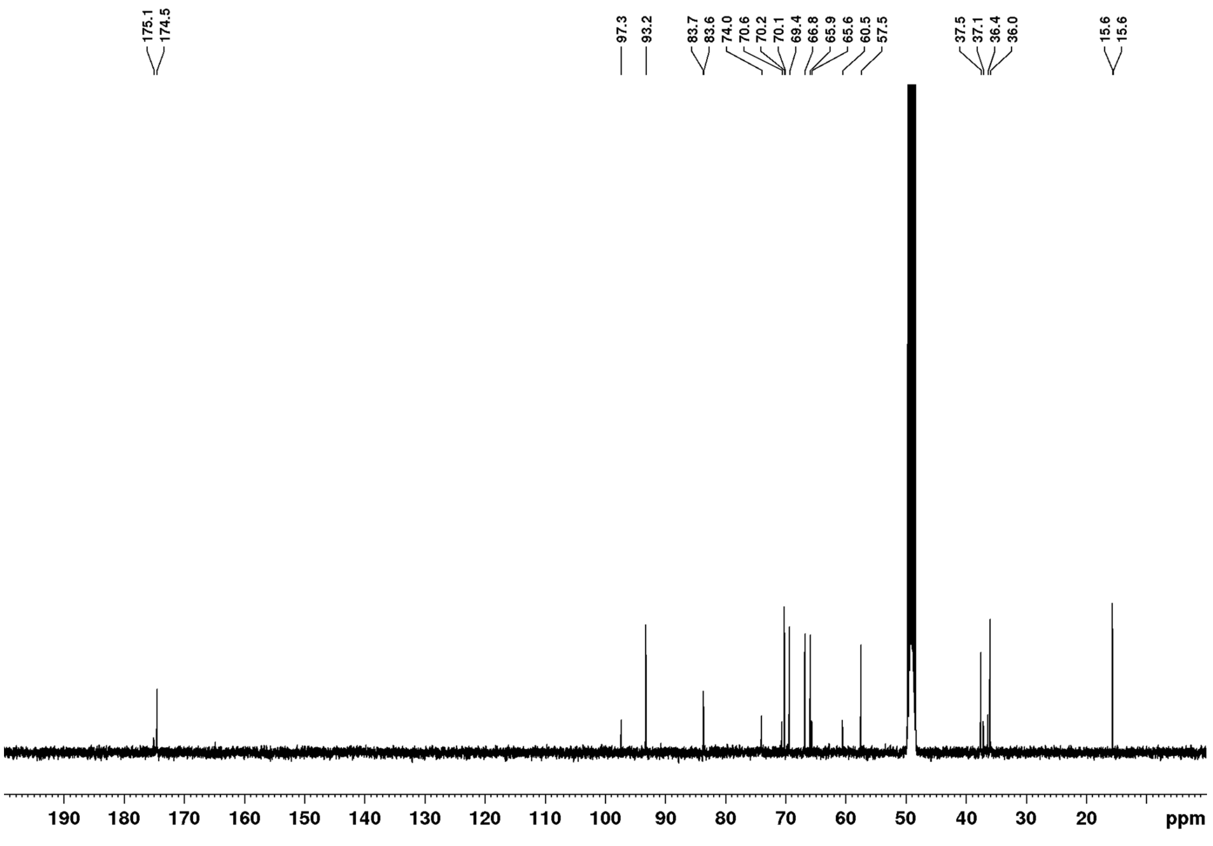

^{1}H NMR (400 MHz, CD_3OD) and ^{13}C NMR (100 MHz, CD_3OD) spectra of compound **3**.

Suppl.Fig.S2

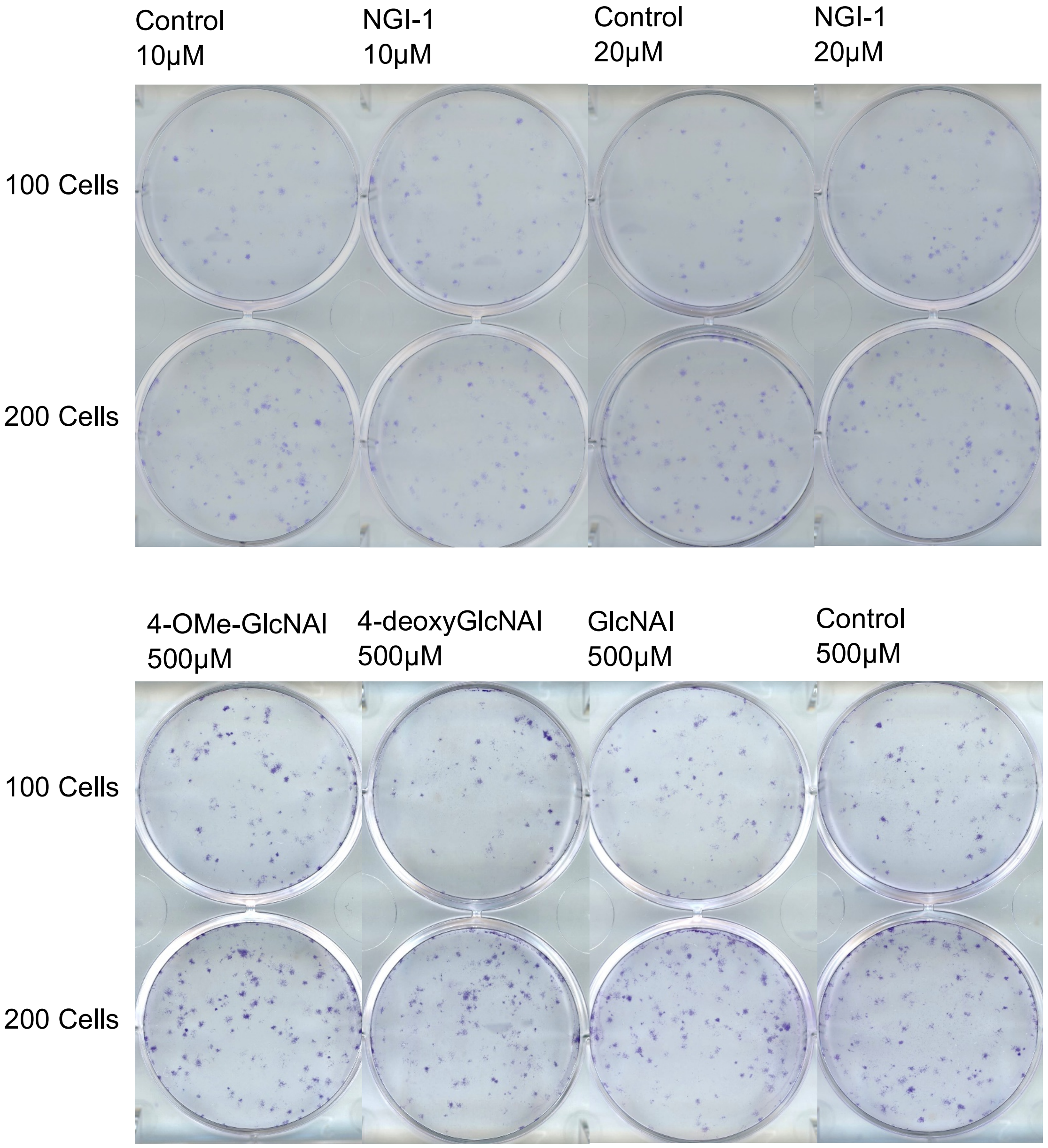
Control
10μM
NGI-1
10μM
Control
20μM
NGI-1
20μM
100 Cells
200 Cells
4-OMe-GlcNAl
500μM
4-deoxyGlcNAl
500μM
GlcNAl
500μM
Control
500μM
100 Cells
200 Cells
